# Comparative analysis of isokinetic ratios between hamstrings and quadriceps in wrestlers: Resting state versus post-fatigue evaluation

**DOI:** 10.1371/journal.pone.0342975

**Published:** 2026-06-12

**Authors:** Ali Tatlıcı, Ahmet Kurtoğlu, Muhammed Yılmaz, Halil Güngör, Ömer Özer, Bekir Çar, Jarosław Muracki, Robert Trybulski, Monira I. Aldhahi

**Affiliations:** 1 Department of Physical Education and Sport Teaching, Faculty of Sport Sciences, Selcuk University, Konya, Türkiye; 2 Department of Coaching Education, Faculty of Sport Science, Bandirma Onyedi Eylul University, Balıkesir, Türkiye; 3 Department of Coaching Education, Faculty of Sport Sciences, Selcuk University, Konya, Türkiye; 4 Department of Physical Education and Sport Teaching, Faculty of Sport Sciences, Selcuk University, Konya, Türkiye; 5 Department of Physical Education and Sport Teaching, Faculty of Sport Sciences, Bandırma Onyedi Eylül University, Balıkesir, Türkiye; 6 Department of Physical Education and Sport Teaching, Faculty of Sport Sciences, Bandırma Onyedi Eylül University, Balıkesir, Türkiye; 7 Institute of Physical Culture Sciences, Department of Physical Culture and Health, University of Szczecin, Szczecin, Poland; 8 Medical Department, Wojciech Korfanty Upper Silesian Academy, Katowice, Poland; 9 Department of Rehabilitation Sciences, College of Health and Rehabilitation Sciences, Princess Nourah bint Abdulrahman University, Riyadh, Saudi Arabia; Erzurum Technical University: Erzurum Teknik Universitesi, TÜRKIYE

## Abstract

**Introduction:**

Wrestling demands high-intensity competition, and tournament structures often require athletes to compete in multiple matches in a single day. This results in accumulated neuromuscular fatigue that may disrupt the balance between hamstring and quadriceps strength, potentially increasing the risk of knee injuries. Therefore, this study aimed to determine whether the conventional hamstring-quadriceps ratio (H: Q_Conv_) and functional hamstring-quadriceps ratio (H: Q_Func_) are more indicative in resting and post-fatigue states, examine the H: Q_Conv_ and H: Q_Func_ isokinetic strength ratios, and analyze the contributions of hamstring and quadriceps strength changes to these ratios.

**Materials and methods:**

The study involved 16 wrestlers who achieved national championship rankings. Their average age was 15.0 ± 1.2 years, with an average height of 171.6 ± 8.6 cm and weight of 65.1 ± 16.6 kg. Their BMI was 21.8 ± 4.0 kg/m², and they had an average of 4.3 ± 1.0 years of training experience. The athletes participated in isokinetic assessments both at rest and after fatigue. The concentric and eccentric strengths of the hamstrings and concentric strength of the quadriceps were measured at angular velocities of 60°/s and 180°/s, respectively.

**Results:**

Two-factor repeated-measures ANOVA revealed a highly significant main effect of time on quadriceps concentric (Q_CON_)(F_(1,15)_=106.789, p < .001, η²ₚ = .877), and angular velocity and interaction were not significant. For H_CON_, there was no time effect, but the velocity effect was significant (60°/s > 180°/s; F_(1,15)_=31.771, p < .001, η²ₚ = .679). For hamstring eccentric (H_ECC_), the time and interaction effects were insignificant, and the velocity remained at the threshold (F_(1,15)_=3.654, p = .075). The H: Q_Conv_ ratio generally increased after fatigue (F = 17.492, p = .001), and there was a level difference between velocities (F = 9.147, p = .009), but the interaction was insignificant. Both time (F = 59.489, p < .001) and velocity (F = 6.716, p = .020) were significant for the H: Q_Func_ ratio, as was the time × velocity interaction (F = 4.708, p = .046); the increase was particularly reliable at 60°/s (Δ=+0.131, 95% CI [0.044, 0.218], p = .006). In the component decompositions, the increase in the time effect was shared similarly by the hamstrings and quadriceps, whereas the hamstrings’ contribution significantly drove the angular velocity difference.

**Conclusion:**

Fatigue significantly reduced the concentric torque of the quadriceps, whereas the concentric/eccentric outputs of the hamstrings were relatively preserved; consequently, the H: Q balance increased, particularly at 60°/s. The time effect was strong for H: Q_Func_, and a time×velocity interaction was present: the increase was reliable at 60°/s but not statistically confirmed at 180°/s. Component decomposition showed that the time-related change was shared to a similar extent by both muscles, whereas the hamstring contribution predominantly explained the angular velocity difference.

## Introduction

Wrestling is regarded as one of the most demanding Olympic events, consisting of repeated short-term high-intensity efforts [1]. This highly competitive environment has physiological consequences, such as lactate accumulation in muscles, disturbance of acid-base balance, and strength losses that primarily affect power in the lower extremity muscle groups [2]. Fatigue during sports activities decreases the potential for force production by lower extremity muscles [3], adversely influences isokinetic performance, and predisposes athletes to strength imbalances around the knee joint [4]. Excessive muscle fatigue can impair hamstring-quadriceps (H: Q) balance and neuromuscular control, thereby reducing knee stability. This condition increases the risk of valgus angulation with sudden “buckling” reactions during dynamic movements, such as landing, changing direction, or making a move, thus contributing to the risk of injury [5]. Knee injuries are among the most common injuries in wrestling and have been reported to account for 26.1% of injuries among wrestlers in the United Kingdom [6]. Furthermore, it has been reported that competition injury rates are approximately 40 times higher than training injury rates, with knee injuries capable of keeping an athlete out of competition for weeks [7].

Knee injuries, especially anterior cruciate ligament injuries, are said to happen about 60% of the time in non-contact ways. These injuries are often linked with quick stops, fast turns, and jumping-landing activities that put tremendous stress on the knee joint [8]. In this situation, the hamstring-to-quadriceps strength ratio is considered an essential neuromuscular factor that affects knee stability. In Greco-Roman wrestling, attacks below the waist are prohibited, which limits direct lower-extremity contact [9]. In contrast, freestyle wrestling allows contact with the legs and lower extremities [9], making contact-related mechanisms more prevalent. Therefore, non-contact injury mechanisms are considered more likely to contribute to knee loading patterns observed in Greco-Roman wrestling.

The H: Q strength ratio can be calculated through various approaches, reflecting different dimensions of knee muscle performance [10], and it has been proposed as a potential protector against hamstring strains and anterior cruciate ligament injuries [11]. Since the H: Q ratio is an indicator of critical importance for maintaining knee joint stability, it is commonly used as one of the parameters to assess the risk for lower extremity injuries [12,13]. The conventional H: Q ratio (H: Q_Conv_) is calculated from the concentric peak torques of the hamstring and quadriceps muscles. It reflects general agonist–antagonist strength balance and is usually assessed at low to intermediate angular velocities [10]. The H: Q_Conv_ strength ratio is about 0.66 [13].

On the other hand, functional H: Q ratio (H: Q_Func_) is a functional measure used in injury risk assessment that describes the relationship between eccentric hamstring and concentric quadriceps peak torques [10]. The H: Q_Func_ ratio is generally considered to be at least 1.0 or more [14]. High-speed running can be regarded as a primary mechanism of hamstring strain [15]. These injuries usually take place in the last part of the swing phase when the hamstring muscles have to produce a lot of force while lengthening [16]. Moreover, it is the co-contraction of the hamstring muscles that prevents excessive contraction of the quadriceps, thus increasing stability at the level of the knee joint and possibly attenuating excessive knee abduction moments during landing tasks [17]. Given these traits, the H: Q_Func_ ratio is seen as offering an evaluation more akin to the mechanical conditions under which knee injuries happen [10]. It has also been proposed that this ratio might be a better measure when assessing movements specific to a sport [18]. These H: Q ratios have become common in professional teams as part of performance assessments and injury risk evaluations [3]. In wrestling, the H: Q ratio is an essential parameter for assessing performance, balance in muscle strength, and injury risk [19,20].

A review of the literature reveals that most strength assessments are performed under resting conditions [21]. Özbay and Ulupınar [22] noted the limitation of this approach by reporting that resting tests could not distinguish performance differences. At the same time, measurements taken after fatigue clearly revealed distinctions between elite and super-elite wrestlers. Similarly, Machado and Nakamura [3] and Pinto and Blazevich [4] emphasized that evaluating parameters such as the H: Q ratio only under resting conditions does not accurately reflect injury risk. In fact, Liu and Peng [23] reported that fatigue might have a significant adverse effect on muscle strength and motor control, leading to imbalances in the knee joint, which increases the risk of ACL and hamstring injuries. It has also been shown that in isokinetic fatigue tests, hamstring muscles are more adversely affected than quadriceps muscles [24]. Other studies have similarly demonstrated a significant decrease in the H: Q ratio with increasing fatigue [25]. The tournament format of wrestling requires athletes to compete in up to five matches within one day, with very short recovery intervals between these matches, resulting in the rapid accumulation of muscle fatigue [26]. Increased fatigue therefore causes disproportionate strength loss between the quadriceps and hamstrings, disrupting the H: Q ratio, reducing knee joint stability, and increasing the risk of injury [3,4]. To the best of our knowledge, the existing literature does not report injury incidence by match order or tournament stage in wrestling competitions. Assessments conducted under fatigue conditions in wrestling can provide more realistic sport-specific insights into an athlete’s capacity for muscle strength, H: Q ratio, and endurance profiles of the hamstring and quadriceps beyond what can be obtained from resting tests. Therefore, the present study aimed to examine the profiles of muscle strength around the knee joint among Greco-Roman wrestlers by evaluating the ratios of H: Q_Conv_ and H: Q_Func_ under both resting and post-fatigue conditions, in addition to determining the relationships between these parameters and injury risk.

## Materials and Methods

### Study design and participants

A crossover design was employed, involving 16 male Greco-Roman wrestlers from the Konya Athlete Training Center (SEM), all of whom were actively engaged in regular wrestling training and volunteered to participate as national-level competitors. The athletes were classified according to the McKay Participant Classification Framework [27] as Tier 3 Highly Trained National-Level athletes, based on their participation. The participants’ mean age was 15.0 ± 1.21 years, height 171.6 ± 8.6 cm, body weight 65.1 ± 16.6 kg, and body mass index (BMI) 21.8 ± 4.0 kg/m². Their average training experience was 4.3 ± 1.0 years, and they were actively engaged in regular Greco-Roman wrestling training programs. The weight category was not controlled as a separate analytical variable. The wrestlers were in the preparation phase before the league season began. The athletes were instructed to avoid any food (except water) for at least 1 hour before the assessments. None of the participants had experienced any musculoskeletal injury or condition affecting lower-limb function for at least 3 months prior to the study. All wrestlers were familiar with the isokinetic knee extension and flexion tests, as they had performed similar assessments in previous seasons. All athletes completed the isokinetic evaluations, and no adverse events were reported during the testing. All participants and their legal guardians were informed about the study procedures, and written informed consent was obtained before their participation. The study protocol was approved by the Selçuk University Faculty of Sports Sciences Ethics Committee (protocol number 92, 16 July 2024, Ethics Committee of Selcuk University, Faculty of Sports Science, Konya, Turkey). It was conducted in accordance with the principles of the Declaration of Helsinki.

### Procedure

**Fig 1:**
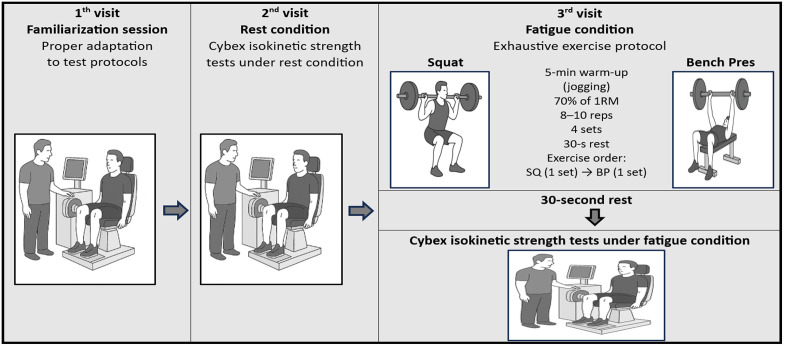
Study design.

This study used an experimental design and was conducted over three separate testing days. On the 1^st^ visit, the participants were familiarized with the study procedures to ensure proper adaptation to the measurement protocol (no strength record). On the 2^nd^ visit, Cybex isokinetic (Cybex NORM®, Humac, CA, USA, 2004) strength tests were performed by athletes under rest conditions, and these measurements were recorded as rest values. All isokinetic strength assessments were performed on the dominant leg of each participant, which was defined as the preferred kicking leg. On the 3^rd^ day, participants underwent an exhaustive exercise protocol, followed immediately by another round of Cybex isokinetic tests (within 30 s). The measurements were recorded as fatigue values. All testing sessions took place between 09:00 and 11:00 a.m., under controlled testing conditions (same testing time, identical equipment setup, standardized procedures, and conducted by the same investigator), with a 48-hour rest period between each testing day to allow for full recovery. Thus, all measurements for each athlete were obtained on three separate days. This design permitted comparisons of muscle strength profiles and H: Q ratios under both rest and fatigue conditions, which are more representative of actual wrestling-specific fatigue, thereby providing more practical insights into performance and injury risk (Fig 1).

### Fatigue-Inducing Exercise Protocol

All subjects were standardized to a similar level of fatigue using an exercise protocol. A simulated wrestling match was avoided for this purpose, given that any variation between wrestlers in their individual tactics and techniques, whether attacking, defending, or applying different holds, could result in unequal levels of fatigue. Therefore, squat (SQ) and bench press (BP), which involve large muscle groups in the lower and upper body, respectively, were selected to predominantly activate the anaerobic energy systems that are heavily utilized in wrestling bouts. Each subject completed four sets at a load equivalent to 70% of their one-repetition maximum (1RM), with 8–10 repetitions for both SQ and BP. The exercises alternated, with one set of SQ followed immediately by one set of BP. Passive rest was allowed for 30 s between all sets and exercises performed under this protocol. The athletes completed a 5-minute jog warm-up prior to the start of the protocol and were verbally encouraged during the exercise period to achieve maximum effort [22].

### Isokinetic Strength Measurement

The strength of the quadriceps and hamstring muscles was determined using a Cybex isokinetic dynamometer (Cybex NORM®, Humac, CA, USA, 2004) in the Kinanthropometry Laboratory at Selçuk University Sports Science Faculty. Participants performed a standardized five-minute warm-up on a cycle ergometer prior to testing at a pedaling cadence of 60–70 revolutions per minute to prepare for the isokinetic evaluation. All participants underwent familiarization consisting of three submaximal repetitions at angular velocities of 60°/s and 180°/s in both concentric and eccentric modes before the actual testing session to ensure adaptation to the protocol [28]. These angular velocities were selected because they are widely used in the literature to assess H: Q strength balance at low and moderate movement speeds [29]. The order of the angular velocity and contraction mode testing was randomized for each participant using a Latin square design. During measurement, subjects were positioned adequately on the dynamometer chair with straps across the thighs and trunk, secured against any extraneous movement. They were also instructed to hold the chair’s lateral handles for stabilization during tests [30,31]. The lever arm length was individually adjusted to the resistance pad just proximal to the medial malleolus, and the contralateral leg was hanging freely without an external load. The axis of the knee joint was aligned with the rotation axis of the dynamometer lever arm at the distal end of the lateral femoral condyle [14]. Concentric muscle torque for quadriceps and hamstrings was measured by three maximal contractions in concentric-concentric mode at 60°/s over a range from 90° to 10° knee flexion, similarly, eccentric torque with three maximal contractions in eccentric-eccentric mode moving from 10° to 90°. The same protocol was repeated at an angular velocity of 180°/s. A passive rest interval of two minutes between test conditions minimized fatigue [32]. Standardized verbal encouragement was provided throughout the procedure to achieve maximal effort, along with real-time visual feedback displayed on a computer screen. For each contraction type, the peak torque (the single highest value across three reps) was recorded and used for statistical analysis; the highest obtained value was considered [30].

### Statistical Analyses

All analyses were conducted using R (v4.x). First, a 2 × 2 repeated measures ANOVA was applied for the variables (Q_CON_, H_CON_, H_ECC_, H: Q_Conv_, H: Q_Func_) to test the main effects of time (rest–fatigue) and angular velocities (60°/s–180°/s) and the time ×Angular Velocity interaction. ANOVA results were reported using F, p, and partial eta-squared (η² p). To clarify the interpretation of the interaction, simple main effects were tested using emmeans following RM-ANOVA (Speed | Time and Time | Speed), comparisons were corrected using Holm’s method, and results were reported using Cohen’s paired d (dᶻ) with 95% CI. The magnitude of the effect sizes was interpreted as follows: η²p = 0.01 (small), 0.06 (medium), and 0.14 (large) [33]. Assumptions were checked at the data/residual level: normality (Shapiro–Wilk), outlier checks (±3 SD), and effect diagnostics. The significance threshold was set at α = 0.05. For sensitivity purposes, torques were additionally normalized according to body mass (Nm·kg ⁻ ¹) and analyzed allometrically as T/mass^b (b estimated via log–log regression in the sample); repeated measures ANCOVA (simple effects adjusted for mass using emmeans) were also conducted, including mass as a covariate.

Figures (rainclouds) show raw data points, density (half-violin), the mean, and the 95% confidence interval (CI) for each condition. To show simple effects, matched comparisons by angular velocity (post–rest; 60–180) were performed, and the mean difference (Δ), 95% confidence interval, p, and matched Cohen’s d (dᶻ) were calculated. Log-ratio decomposition was applied to examine which component drove the H: Q ratios: for H: Q_conv_, Δlog(H_CON_) and −Δlog(Q_CON_); for H: Q_Func_, Δlog(H_ECC_) and −Δlog(Q_CON_) were calculated. For the contrasts of time (resting → fatigue) and angular velocities (60 → 180), within-subject contributions were obtained, and the difference in the contribution of the quadriceps and hamstrings was tested using a paired t-test and reported with dᶻ and 95% CI. The packages ggplot2/ggdist were used for visualization. All tests were two-tailed, and summaries were presented as mean ± SD (or Δ for ratios/contributions, 95% CI).

## Results

Table 1 presents the mean and standard deviation values of the quadriceps and hamstring torques, as well as the H: Q_Conv_ and H: Q_Func_ ratios, measured at 60°/s and 180°/s under pre- and post-fatigue conditions.

**Table 1:** Descriptive statistics of quadriceps and hamstring torque values and hamstring-to-quadriceps (H: Q) ratios at 60°/s and 180°/s under rest and fatigue conditions.

Angular velocity (°/s)	Time	Q_CON_ (Nm)	H_CON_ (Nm)	H_ECC_ (Nm)	H: Q_Conv_	H: Q_Func_
60°/s	Rest	131.75 ± 41.78	101.56 ± 33.43	110.56 ± 35.34	0.77 ± 0.06	0.84 ± 0.07
	Fatigue	122.13 ± 32.10	105.81 ± 29.43	116.56 ± 29.11	0.87 ± 0.15	0.97 ± 0.17
180°/s	Rest	95.69 ± 31.80	84.50 ± 25.23	108.56 ± 29.71	0.90 ± 0.13	1.17 ± 0.20
	Fatigue	92.69 ± 26.75	86.69 ± 24.41	109.06 ± 26.33	0.94 ± 0.11	1.20 ± 0.18

Q_CON_: Quadriseps concentric, H_CON_: Hamstring concentric, H_ECC_: Hamstring eccentric.

Table 2 presents the results of the two-factor repeated-measures ANOVA for Time (pre–post) × Velocity (60°/s–180°/s). The main effect of time for Q_CON_ was very large (F_(1,15)_=106.789, p < .001, η²ₚ = .877), whereas the main effect of velocity (F_(1,15)_ =2.034, p = .174, η²ₚ = .119) and the interaction (F_(1,15)_=2.545, p = .132, η²ₚ = .145) were not significant. There was no time effect in H_CON_ (F_(1,15)_=0.833, p = .376, η²ₚ = .053), but the main effect of angular velocity was significant (F_(1,15)_=31.771, p < .001, η²ₚ = .679); and the interaction was insignificant (F_(1,15)_=0.458, p = .509, η²ₚ = .030). Neither time (F(1,15)=0.751, p = .400, η²ₚ = .048) nor interaction (F_(1,15)_=2.604, p = .127, η²ₚ = .148) were significant for H_ECC_; the angular velocity effect was at the trend level (F_(1,15)_=3.654, p = .075, η²ₚ = .196). Both the main effects of time (F_(1,15)_=17.492, p = .001, η²ₚ = .538) and angular velocity (F_(1,15)_=9.147, p = .009, η²ₚ = .379) were significant for the H: Q_Conv_ ratio, but the interaction was not significant (F_(1,15)_=2.079, p = .170, η²ₚ = .122). The main effects of time (F_(1,15)_=59.489, p < .001, η²ₚ = .799) and velocity (F_(1,15)_=6.716, p = .020, η²ₚ = .309) on the H: Q_Func_ ratio, as well as the Time × Velocity interaction, were significant (F_(1,15)_=4.708, p = .046, η²ₚ = .239). We additionally tested simple main effects (Holm-adjusted) of Velocity within each level of Time and of Time within each Velocity. For Q_CON_, the within-speed time contrasts reproduced the same direction but did not reach significance at either speed (60°/s: Rest−Post = +9.62 Nm, 95% CI [−2.79, 22.00], p = .119; 180°/s: + 3.00 Nm, [−4.95, 11.00], p = .434). For H_CON_, Velocity was significantly higher at 60°/s than 180°/s both at rest and post-fatigue (Rest: + 17.1 Nm, [+10.8, + 23.3], p < .001; Post: + 19.1 Nm, [+10.4, + 27.8], p < .001), whereas the Time simple effects were not significant within either speed (60°/s: Rest−Post = −4.25 Nm, [−12.3, 3.84], p = .281; 180°/s: −2.19 Nm, [−10.5, 6.10], p = .582). For HECC, the Velocity simple effect was significant only post-fatigue (Post: + 7.5 Nm, [+1.81, + 13.19], p = .013), with no significant time contrasts within speeds. For H: Q_Conv_, the Time simple effect was significant at 60°/s(Rest−Post = −0.104, [−0.174, −0.034], p = .006), but not at 180°/s (−0.042, [−0.109, 0.025], p = .201); the Velocity simple effect was significant at rest (−0.132, [−0.185, −0.078], p < .001). For H: Q_Func_, the Time×Velocity interaction was clarified by a significant time contrast at 60°/s (Rest−Post = −0.131, [−0.217, −0.044], p = .006) but not at 180°/s(−0.035, [−0.114, 0.043], p = .356), while the Velocity simple effects were significant at both time levels (Rest: −0.329, [−0.430, −0.228], p < .001; Post: −0.233, [−0.312, −0.154], p < .001). These simple-effects results make explicit how velocity behaves within rest and post-fatigue and confirm the omnibus ANOVA conclusions.

**Table 2:** Results of the 2 × 2 repeated-measures ANOVA of quadriceps and hamstring strength.

Parameter	Effect	F_(1,15)_	p	η²ₚ
**Q**_CON_ (Nm/kg^-1^)	Time	106.789	<.001***	.877
	Velocity	2.034	.174	.119
	Time × Velocity	2.545	.132	.145
**H**_**CON**_ (Nm/kg^-1^)	Time	0.833	.376	.053
	Velocity	31.771	<.001***	.679
	Time × Velocity	0.458	.509	.030
**H**_**ECC**_ (Nm/kg^-1^)	Time	0.751	.400	.048
	Velocity	3.654	.075	.196
	Time × Velocity	2.604	.127	.148
**H: Q**_**Conv**_	Time	17.492	.001**	.538
	Velocity	9.147	.009**	.379
	Time × Velocity	2.079	.170	.122
**H: Q**_**Func**_	Time	59.489	<.001***	.799
	Velocity	6.716	.020*	.309
	Time × Velocity	4.708	.046*	.239

Note. Velocity: angular velocity, the asterisk (*) indicates a statistically significant difference (*p <.05, **p <.01, **p <.001).

**Fig 2:**
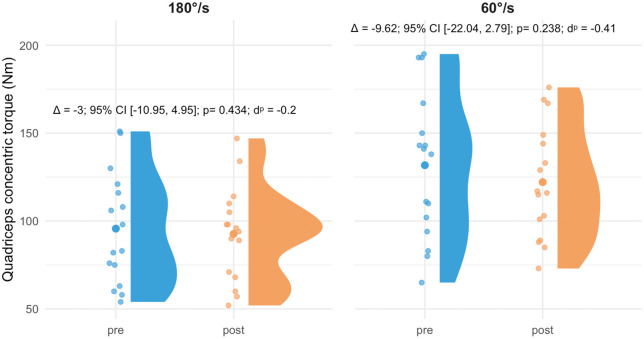
Raincloud plot of resting (pre) and post-fatigue values for the concentric torque of the quadriceps (Q_CON_) at 60°/s and 180°/s: The points represent individual observations, the half-violin distribution represents the sample distribution, the horizontal lines represent group means, and the short lines above them represent the 95% confidence interval.

Fig 2 shows that post-fatigue concentric torque of the quadriceps tends to be lower than the pre-fatigue state for both 60°/s and 180°/s; however, the difference is not statistically significant as the confidence intervals include zero (60°/s: Δ=−3.00 Nm, 95% CI [−10.95, 4.95], p = .434, d=−0.20; 180°/s: Δ=−9.62 Nm, 95% CI [−22.04, 2.79], p = .238, d=−0.41). This pattern indicates a general decrease at both angular velocities, and the main effect of Time in the ANOVA test results was very large and significant, consistent with the results that the main impact of velocity and the Time×Angular Velocity interaction were not significant. That is, the fatigue-reducing effect on Q_CON_ appears to be independent of velocity. However, the differences within each angular velocity in the figure do not individually cross the statistical threshold (because the CIs include zero). When all conditions are considered together, the omnibus time effect is strongly detected (particularly because Holm correction and smaller samples split by angular velocity may have prevented significance in simple effects). Practically, a similar decrease in the concentric capacity of the quadriceps muscle after fatigue should be expected at both angular velocities; velocity does not significantly moderate this decrease.

**Fig 3:**
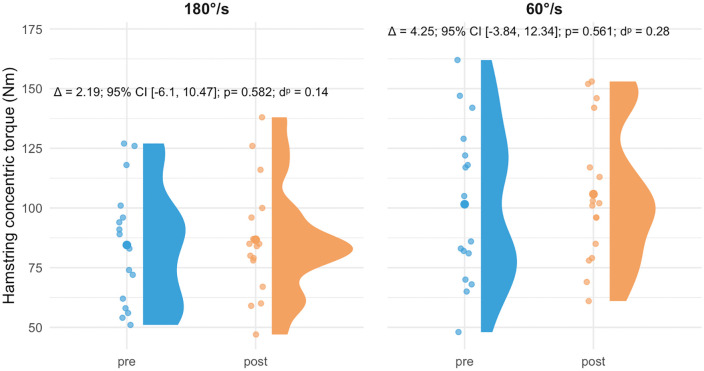
Raincloud plot of resting (pre) and post-fatigue values for the concentric torque of the hamstring (H_CON_) at 60°/s and 180°/s: The points represent individual observations, the half-violin distribution represents the sample distribution, the horizontal lines represent group means, and the short lines above them represent the 95% confidence interval.

As shown in Fig 3, at 180°/s, fatigue showed a small and insignificant increase compared to the resting state (Δ=+2.19 Nm; 95% CI [−6.10, 10.47]; p = .582; d ≈ 0.14). Similarly, there was a small and non-significant increase at 60°/s (Δ=+4.25 Nm; 95% CI [−3.84, 12.34]; p = .561; d ≈ 0.28). These pairwise comparisons are consistent with the findings of the two-factor repeated-measures ANOVA: the main effect of time was not significant, and the time × Angular Velocity interaction was also not significant; therefore, the impact of fatigue on H_CON_ remained small and similar across angular velocities. In contrast, the main effect of angular velocity was strong, confirming the tendency for absolute torque levels at 60°/s to be significantly higher than those at 180°/s. The inclusion of zero in the confidence intervals and the insignificance of the time/interaction terms indicate that the H_CON_ changes after fatigue in this sample are clinically small and statistically uncertain. In practice, this suggests that hamstring concentric performance is angular-velocity-dependent (60°/s > 180°/s) but relatively resistant to fatigue; therefore, while angular-velocity differences should be considered in monitoring/recovery strategies, short-term H_CON_ changes post-fatigue alone should not be decisive.

**Fig 4:**
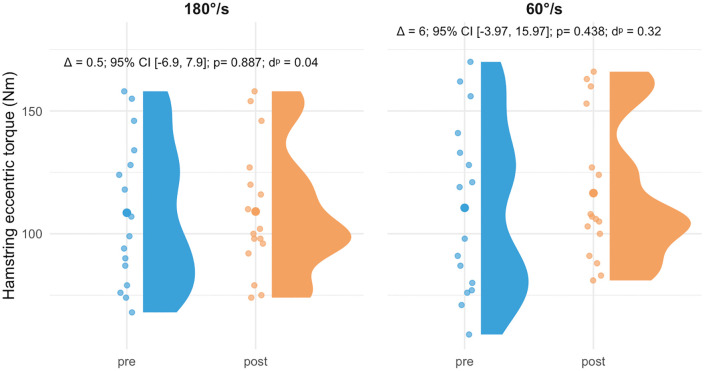
Raincloud plot of resting (pre) and post-fatigue values for the eccentric torque of the hamstring (H_ECC_) at 60°/s and 180°/s: The points represent individual observations, the half-violin distribution represents the sample distribution, the horizontal lines represent group means, and the short lines above them represent the 95% confidence interval.

The two-factor repeated-measures ANOVA indicated that the main effect of time was not significant for H_ECC_, the main effect of Angular Velocity remained at the statistical limit, and the Time×Angular Velocity interaction was not important. The paired comparisons reported in Fig 4 are consistent with this table: at 180°/s, post-fatigue torque showed a very small and unreliable increase compared to the resting condition (Δ=+0.50 Nm; 95% CI [−6.90, 7.90]; p = .887; d ≈ 0.04); the increase at 60°/s appeared larger but was not statistically significant (Δ=+6.00 Nm; 95% CI [−3.97, 15.97]; p = .438; d ≈ 0.32). The inclusion of zero in the confidence intervals and the lack of significance for both the time and interaction terms in the ANOVA suggest that hamstring eccentric performance is generally robust against the fatigue protocol; possible angular velocity differences did not reach the level of evidence in this study. In practical terms, a significant deterioration in eccentric hamstring torque should not be expected in post-fatigue testing; the slight increase observed at 60°/s should be interpreted clinically while considering the statistical uncertainty.

**Fig 5:**
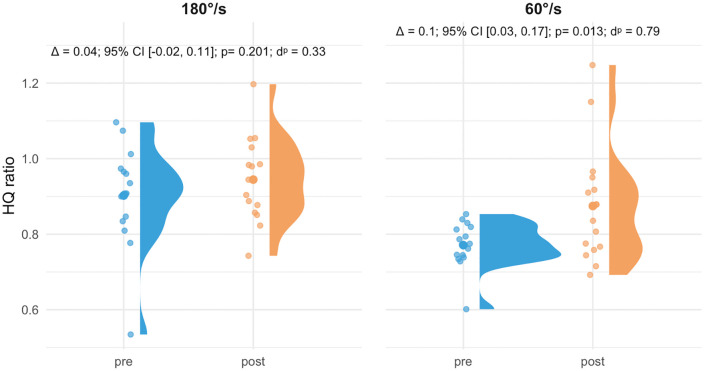
Raincloud plot of resting (pre) and post-fatigue values for the H: Q_Conv_ at 60°/s and 180°/s: The points represent individual observations, the half-violin distribution represents the sample distribution, the horizontal lines represent group means, and the short lines above them represent the 95% confidence interval.

Fig 5 shows that the post-fatigue ratio at 60°/s in the H: Q_Conv_ group exhibited a significant and relatively large increase compared to the resting state (Δ=+0.10; 95% CI [0.03, 0.17]; p = .013; d ≈ 0.79). In contrast, the post-fatigue increase at 180°/s was slight and statistically uncertain (Δ=+0.04; 95% CI [−0.02, 0.11]; p = .201; d ≈ 0.33). The fact that the confidence interval at 60°/s does not include zero indicates a reliable improvement in the H: Q_Conv_ balance from rest to post-fatigue, whereas the inclusion of zero at 180°/s suggests that this difference cannot be demonstrated at the sample level at high angular velocity. This pattern of figures indicates that there is a general increase after fatigue (main effect of Time: F(1,15)=17.492, p = .001, η²ₚ = .538) and a general difference in level between angular velocities (Velocity: F_(1,15)_=9.147, p = .009, η²ₚ = . 379); however, it supports that the increase did not differ significantly according to velocity (Time×Velocity: F_(1,15)_=2.079, p = .170, η²ₚ = .122). In practice, this indicates that the post-fatigue conventional H:Q balance increases in a clinically significant manner relative to the resting state, particularly at a low angular velocity (60°/s), whereas the change remains small and uncertain at a high angular velocity (180°/s). This indicates that, while the general trend of balance improvement from rest to post-fatigue remains independent of angular velocity, its magnitude is greater at 60°/s. In this context, an increase in the H: Q_conv_ ratio reflects a change in the relative contributions of the hamstrings and quadriceps; therefore, “improvement” mainly results from Q_CON_ (the denominator) decreasing more with fatigue, while H_CON_ (the numerator) is less affected/relatively preserved. In other words, an increase in the ratio does not necessarily mean that the hamstrings have increased in absolute terms; in most cases, the decrease in the quadriceps is greater than that in the hamstrings. Therefore, the relative balance shifts in favor of hamstrings. This pattern demonstrates that the direction of the balance shift during the transition from rest to fatigue is determined by the differing sensitivities of the two muscle groups, with the increase essentially reflecting the differential fatigue effect.

**Fig 6:**
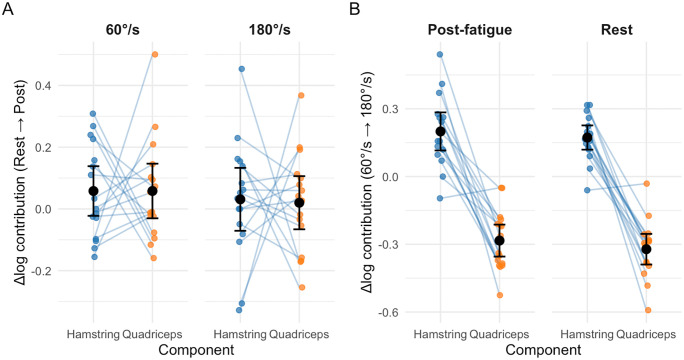
Separation of component contributions in the H: Q_conv_ ratio on a log scale: Panel 6A: Separated representation of the H: Q_Conv_ change from rest to post-fatigue (Rest → Post) into hamstring (ΔlogH) and quadriceps (−ΔlogQ) contributions at each angular velocity H: Q_Conv_°/s, 180°s). Panel 6B: Effect of angular velocity difhamstring60°/s → 180°/quadricepsonv, decomposed into Hamstring and Quadriceps contributions for each time level (Rest, Post-fatigue).

Fig 6A shows the distribution of contributions to the change from rest to post-fatigue according to angular velocities, and the contributions of the hamstrings (ΔlogH) and quadriceps (−ΔlogQ) appear to be of equal magnitude at both angular velocities; therefore, there is no evidence that a single muscle predominantly drives the increase in H: Q_Conv_ observed at 60°/s post-fatigue (60°/s: hamstring − quadriceps = 0.000, 95% CI [−0.151, 0.151]; p = .999; dᶻ = 0.00; 180°/s: 0.011, [−0.162, 0.184]; p = .894; dᶻ = 0.03).

Fig 6B shows which component accounts for the difference in angular velocity between 60°/s and 180°/s, separately for at rest and after fatigue; here, the hamstring contribution is significantly higher, and the differences are consistently above zero with wide confidence intervals (Post-fatigue: Hamstring − quadriceps = 0.483, 95% CI [0.353, 0.613]; p < .001; dᶻ = 1.98. Resting: 0.494, [0.389, 0.600]; p < .001; dᶻ = 2.50). This pattern indicates that both muscles contribute similarly to the time effect, but the hamstrings are dominant in the angular velocity effect.

**Fig 7:**
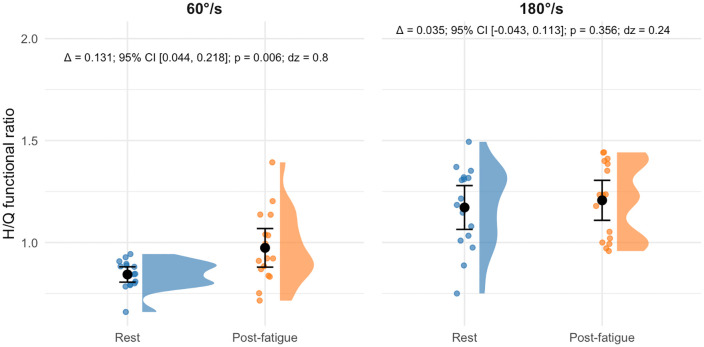
Raincloud plot of resting (pre) and post-fatigue values for the H: Q_Func_ at 60°/s and 180°/s: The points represent individual observations, the half-violin distribution represents the sample distribution, the horizontal lines represent group means, and the short lines above them represent the 95% confidence interval.

Fig 7 shows that the functional H: Q ratio increased significantly at 60°/s compared to the resting state after fatigue; however, the change remained small and unreliable at 180°/s (60°/s: Δ=+0.131; 95% CI [0.044, 0.218]; p = 0.006; dᶻ = 0.80; 180°/s: Δ=+0.035; 95% CI [−0.043, 0.113]; p = 0.356; dᶻ = 0.24). This figure pattern is consistent with the two-factor repeated-measures ANOVA results: the main effect of time is strong, with post-fatigue > rest, and there is also a general level difference between angular velocities. Notably, the Time × Angular Velocity interaction was significant. In this case, the enhancing effect of fatigue was evident and reliable at 60°/s but could not be statistically confirmed at 180°/s (the confidence interval included zero). Therefore, it can be said that the increase in functional H: Q balance arises from the effect of time; however, its magnitude varies with angular velocity and is particularly visible at 60°/s.

**Fig 8:**
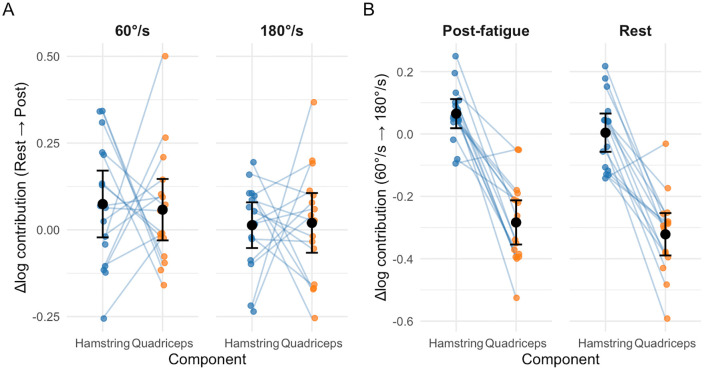
Separation of component contributions in the H: Q_Conv_ ratio on a log scale: Panel A: Separated representation of the H: Q, _Conv_ change from rest to post-fatigue (Rest → Post) into Hamstring (ΔlogH) and Quadriceps (−ΔlogQ) contributions at each angular velocity (60°/s, 180°/s). Panel B: Effect of angular velocity difference (60°/s → 180°/s) on H: Q_Conv_, decomposed into Hamstring and Quadriceps contributions for each time level (Rest, Post-fatigue).

In Fig 8A, in the transition from rest to post-fatigue, the contributions of the hamstrings (ΔlogH) and quadriceps (−ΔlogQ) appear to be similar in magnitude at both angular velocities; that is, there is no evidence that a single muscle ‘dominantly’ drives the increase in balance observed at 60°/s post-fatigue (60°/s: Δ=+0.016, 95% CI [−0.146, 0.179]; p = 0.833; dz = 0.05; 180°/s:Δ=−0.007, [−0.143, 0.130]; p = 0.919; dz=−0.03). This pattern indicates that the relative contributions of the hamstrings and quadriceps remain balanced.

Fig 8B shows the component responsible for the angular velocity difference between 60°/s and 180°/s separately for rested and post-fatigue conditions. The hamstring contribution was significantly higher at both time points, and the differences were consistently above zero (post-fatigue: Δ=+0.349, 95% CI [0.254, 0.444]; p < .001; dz = 1.96; Rest: Δ=+0.326, [0.232, 0.420]; p < .001; dz = 1.85). This pattern indicates that the hamstrings are dominant in the angular velocity effect; in contrast, for the time effect, both muscles contribute to a similar extent.

## Discussion

The present study examined changes in knee joint muscle strength and the H: Q_Conv_ and H: Q_Func_ ratios in Greco-Roman wrestlers pre- and post-fatigue. Both ratios increased following fatigue, primarily due to a significant decrease in quadriceps strength, whereas hamstring strength remained relatively unchanged. Despite both ratios increasing post-fatigue, the magnitude of change was velocity-dependent, with a significant increase in the H: Q_Func_ ratio at 60°/s but not at 180°/s. Additionally, post-hoc analysis indicated that fatigue affected both muscle groups similarly at both angular velocities, as evidenced by the absence of interaction effects, suggesting that the effects of fatigue were additive. The apparent difference between the angular velocities was attributed to a greater percentage contribution from the hamstrings at 60°/s compared to 180°/s.

Wrestling consists of numerous short, high-intensity efforts performed intermittently within a match or across tournament days [34]. During tournaments, athletes typically perform multiple bouts in a single day, which may result in significant performance decrements [26]. Kraemer *et al.* also described substantial reductions in the strength and explosive power of wrestlers between successive matches on the same day [2]. Fatigue has been associated with impaired muscle function and an increased risk of hamstring strain, particularly in soccer players [35], as well as anterior cruciate ligament injuries [36]. However, no study has directly addressed this issue in wrestling. Wright, Ball, and Wood [37] reported increases in both conventional and functional H: Q ratios following fatigue in soccer players (H:Q_Conv_: pre = 0.75, post = 1.02; H:Q_Func_: pre = 0.88, post = 1.08). They attributed these changes to greater hamstring coactivation during quadriceps contractions under fatigue conditions, indicating that the hamstrings acted as stabilizers to protect the knee joint [37]. Thus, there could be an even greater performance loss for the quadriceps due to its own fatigue and increased antagonist resistance from the hamstrings. In this study, a similar phenomenon seems to explain the increase in the H: Q_Conv_ and H: Q_Func_ ratios after fatigue events. In this study, conducted among youth Greco-Roman wrestlers, a similar phenomenon appears to explain the increase in the H: Q_Conv_ and H: Q_Func_ ratios following fatigue events.

After fatigue, both the H: Q_Conv_ and H: Q_Func_ ratios increased in youth Greco-Roman wrestlers, mainly due to a significant decrease in quadriceps strength, while the hamstrings remained relatively unchanged. This indicates that fatigue had a greater effect on the quadriceps, thereby shifting the balance between the two muscle groups. The logarithmic contribution analysis supported this finding, showing that the quadriceps and hamstrings contributed almost equally to the changes in the ratio with fatigue. These results directly relate to the effect of the fatigue protocol on each of these specific muscle groups. Wrestling actions, such as pushing, lifting, and maintaining an upright combat position, require repetitive activation of the quadriceps [1,38]. The current study protocol, consisting of repeated sets of squats and bench presses at 70% 1RM with short rest periods, primarily stressed the quadriceps and likely led to marked strength reduction in that area, with lesser effects on hamstring strength.

In contrast to the findings in youth Greco-Roman wrestlers in the present study, fatigue has been reported to affect the hamstrings more severely in sports such as soccer, futsal, basketball, and handball, thereby decreasing the H: Q ratio [3,4,24,39–43]. This is because they are highly engaged during sprinting, kicking, and rapid changes in direction, which involve intense eccentric muscle contractions. In addition to having a higher percentage of type II fibers [44], complex muscle architecture, and being biarticular, these characteristics make the hamstrings more vulnerable to fatigue [41]. These physiological characteristics may also help explain why, in the present study, the hamstrings played a leading role in the velocity-related changes in the H: Q ratios identified by logarithmic contribution analysis. Furthermore, this study is among the very few that have applied a logarithmic contribution analysis to examine muscle-specific determinants of H: Q ratio changes under different fatigue and angular velocity conditions.

The exercise protocol that causes fatigue used in the present study (squat and bench press at 70% of 1RM with short rest intervals) was previously reported by Özbay and Ulupınar as a method to create wrestling-specific fatigue and has been validated in the literature [22]. It was designed to simultaneously activate major upper- and lower-body muscle groups, thereby engaging anaerobic energy systems similar to those used during wrestling bouts. A simulated match was avoided because wrestlers’ individual strategies and techniques, such as attacking, defending, and grappling, could lead to inconsistent fatigue levels. Instead, a standardized approach ensured that all participants experienced the same degree of fatigue. Post-fatigue protocol testing revealed a significant decrease in quadriceps strength, with no statistically significant change in hamstrings, an expected outcome based on the nature of the protocol, which primarily involved efforts demanding quadriceps support relative to technical and physiological demands in wrestling.

Furthermore, this reduction in performance cannot be attributed purely to peripheral fatigue, as protective mechanisms within the central nervous system can reduce motor neural drive, contributing to additional strength loss during repeated high-intensity efforts [41,45]. This may further explain the decline in quadriceps strength with an increase in the H: Q ratio. In this sense, one could consider the applied protocol a valid and reliable means of inducing wrestling-specific fatigue in suitable model-based research within the field.

In this study, both the H: Q_Conv_ and H: Q_Func_ ratios increased after fatigue. However, only the functional ratio showed a significant time × velocity interaction, indicating greater sensitivity to changes in both fatigue and contraction angular velocity. The effect size analysis revealed a large effect of fatigue on the H: Q_Func_ ratio, whereas the effects observed for the H: Q_Conv_ ratio were comparatively smaller and less sensitive to velocity-related changes. This is consistent with previous studies that identified the H: Q_Func_ ratio as a more robust indicator of neuromuscular balance and injury risk [4]. Croisier *et al*. reported that reduced H: Q_Func_ ratios represent one of the most common neuromuscular imbalances in athletes and are better predictors of injury risk than reduced H: Q_Conv_ ratios [11]. Baroni *et al.* also emphasized that the H: Q_Func_ ratio more accurately reflects sport-specific muscle coordination demands, particularly at higher angular velocities [10]. Overall, the presence of larger, velocity-dependent effects in the Q_Func_ ratio supports its greater practical relevance compared with the conventional ratio. These results highlight the importance of assessing the H: Q_Fun c_ ratio, especially under fatigue and varying angular velocity conditions, as a more informative and practical approach for monitoring muscular balance and injury susceptibility in athletes.

This study has several limitations. First, because of the relatively small sample size, these findings cannot be generalized to the greater wrestling community or athletes from other sports. Another limitation of this study is that the participants were relatively early in their competitive development, which may limit the generalizability of the findings to more experienced or elite adult Greco-Roman wrestlers. Although the fatigue protocol was carefully designed to replicate the metabolic and muscular demands specific to wrestling, it may not have fully captured the technical variability and psychological stress occurring in real matches. In addition, a cross-sectional design cannot establish a direct causal link between post-fatigue changes seen in the H: Q_Conv_ and H: Q_Func_ ratios and the actual risk of injury. Thus, future studies should include larger cohorts and simulate match conditions using longitudinal designs to better verify and expand on the current results. Despite these limitations, this study takes an essential step toward understanding post-fatigue muscle function among youth wrestlers and toward gaining insight into the sport-specific mechanisms of fatigue in this population.

## Conclusion

This study examined the effects of a fatigue-inducing protocol on the muscle strength profiles of youth Greco-Roman wrestlers. They found a significant decrease in quadriceps strength after fatigue, whereas the reduction in hamstring performance was relatively small. Consequently, both the H:Q_Conv_ and H: Q_Func_ ratios increased after fatigue. Of these two ratios, the H: Q_Func_ was more sensitive to changes in angular velocity and, therefore, might be better for capturing high-angular velocity, sport-specific demands in wrestling. In short, the technical and physiological characteristics of wrestling seem to lead to greater fatigue-related declines in the quadriceps, contributing to the observed increases in the H: Q ratios. These results suggest that, particularly at the lower angular velocity of 60 °/s, the HQ_Func_ ratio demonstrated a significant growth and may therefore be a more sensitive measure of fatigue muscle balance as it relates to potential injury risks for wrestlers. Thus, both fatigue and movement velocity should be considered when developing training strategies and injury prevention programs for youth Greco-Roman wrestlers. However, these findings should be interpreted with caution, as they are specific to youth Greco-Roman wrestlers and may not be directly generalizable to adult or elite wrestling populations.

## Supporting information

S1 Dataset Raw data for resting state and post-fatigue evaluations. (XLSX)
