# Supplementary material for: Comparative analysis of isokinetic ratios between hamstrings and quadriceps in wrestlers: Resting state versus post-fatigue evaluation
Source: PLoS One. 2026 Jun 12;21(6):e0342975. doi: 10.1371/journal.pone.0342975 (PMC13262851; doi:10.1371/journal.pone.0342975)

PLOS ONE

Comparative Analysis of Isokinetic Ratios Between Hamstrings and Quadriceps in Wrestlers: Resting State Versus Post-Fatigue Evaluation

--Manuscript Draft--

**Manuscript Number:**	PONE-D-25-60422
**Article Type:**	Research Article
**Full Title:**	Comparative Analysis of Isokinetic Ratios Between Hamstrings and Quadriceps in Wrestlers: Resting State Versus Post-Fatigue Evaluation
**Short Title:**	Isokinetic Ratios of Quadriceps and Hamstrings
**Corresponding Author:**	Ahmet Kurtoğlu<br>Bandirma Onyedi Eylul Universitesi<br>Balıkesir, TÜRKIYE
**Keywords:**	Isokinetic Strength; H:Q Ratio; Muscle Fatigue; Wrestling
**Abstract:**	Introduction<br><br>This study aimed to determine whether the conventional hamstring-quadriceps ratio (H:QConv) and functional hamstring-quadriceps ratio (H:QFunc) are more indicative in resting and post-fatigue states, examine the H:QConv and H:QFunc isokinetic strength ratios, and analyze the contributions of hamstring and quadriceps strength changes to these ratios.<br><br>Materials and methods<br><br>The study involved 16 wrestlers who achieved rankings in national championships. Their average age was 15.0 ± 1.2 years, with an average height of 171.6 ± 8.6 cm and weight of 65.1 ± 16.6 kg. Their BMI was 21.8 ± 4.0 kg/m2, and they had an average of 4.3 ± 1.0 years of training experience. The athletes participated in isokinetic assessments both at rest and after fatigue. The concentric and eccentric strengths of the hamstrings and concentric strength of the quadriceps were measured at angular velocities of 60°/s and 180°/s, respectively.<br><br>Results<br><br>Two-factor repeated-measures ANOVA revealed a highly significant main effect of time on QCON (F(1,15)=106.789, p<.001, η2=.877), and angular velocity and interaction were not significant. For HCON, there was no time effect, but the velocity effect was significant (60°/s > 180°/s; F(1,15)=31.771, p<.001, η2=.679). For HECC, the time and interaction effects were insignificant, and the velocity remained at the threshold (F(1,15)=3.654, p=.075). The H/Qconv ratio generally increased after fatigue (F=17.492, p=.001), and there was a level difference between velocities (F=9.147, p=.009), but the interaction was insignificant. Both time (F=59.489, p<.001) and velocity (F=6.716, p=.020) were significant for the H/Qfunc ratio, as was the time × velocity interaction (F=4.708, p=.046); the increase was particularly reliable at 60°/s (Δ=+0.131, 95% CI [0.044, 0.218], p=.006). In the component decompositions, the increase in the time effect was shared similarly by the hamstrings and quadriceps, whereas the hamstrings' contribution significantly drove the angular velocity difference.<br><br>Conclusion<br><br>Fatigue significantly reduced the concentric torque of the quadriceps, whereas the concentric/eccentric outputs of the hamstrings were relatively preserved; consequently, the H/Q balance increased, particularly at 60°/s. The time effect was strong for functional H/Q, and a time × velocity interaction was present: the increase was reliable at 60°/s but not statistically confirmed at 180°/s. Component decomposition showed that the time-related change was shared to a similar extent by both muscles, whereas the angular velocity difference was predominantly explained by the hamstring contribution.

Powered by Editorial Manager® and ProduXion Manager® from Aries Systems Corporation

Order of Authors:	Ali Tatlıcı
	Ahmet Kurtoğlu
	Muhammed Yılmaz
	Halil Güngör
	Ömer Özer
	Bekir Çar
	Jarosław Muracki
	Robert Trybulski
	Monira I. Aldhahi
Additional Information:	
Question	Response
**Financial Disclosure**<br><br>Enter a financial disclosure statement that describes the sources of funding for the work included in this submission. Review the submission guidelines for detailed requirements. View published research articles from *PLOS ONE* for specific examples.<br><br>This statement is required for submission and **will appear in the published article** if the submission is accepted. Please make sure it is accurate.<br><br>**Funded studies**<br>Enter a statement with the following details:<br>• Initials of the authors who received each award<br>• Grant numbers awarded to each author<br>• The full name of each funder<br>• URL of each funder website<br>• Did the sponsors or funders play any role in the study design, data collection and analysis, decision to publish, or preparation of the manuscript?<br><br>Did you receive funding for this work?	The author(s) received no specific funding for this work.
**Competing Interests**<br><br>Use the instructions below to enter a competing interest statement for this submission. On behalf of all authors, disclose any competing interests that	The authors have declared that no competing interests exist.

could be perceived to bias this work—acknowledging all financial support and any other relevant financial or non-financial competing interests.<br><br>This statement is required for submission and **will appear in the published article** if the submission is accepted. Please make sure it is accurate and that any funding sources listed in your Funding Information later in the submission form are also declared in your Financial Disclosure statement.<br><br>View published research articles from *PLOS ONE* for specific examples.<br><br>**NO authors have competing interests**<br><br>Enter: *The authors have declared that no competing interests exist.*<br><br>**Authors with competing interests**<br><br>Enter competing interest details beginning with this statement:<br><br>*I have read the journal's policy and the authors of this manuscript have the following competing interests: [insert competing interests here]*<br><br>* typeset	
**Ethics Statement**<br><br>Enter an ethics statement for this submission. This statement is required if the study involved:<br><br>• Human participants<br>• Human specimens or tissue<br>• Vertebrate animals or cephalopods<br>• Vertebrate embryos or tissues<br>• Field research<br><br>Write "N/A" if the submission does not require an ethics statement.	All procedures and research protocols in this study involving human participants were approved by the ethical standards of the Institutional Review Board (protocol number 92, 16 July 2024, Ethics Committee of Selcuk University, Faculty of Sports Science, Konya, Turkey). Written Informed consent was obtained from each participant, which was a condition for enrolment in the study, in accordance with the Declaration of Helsinki.

General guidance is provided below. Consult the submission guidelines for detailed instructions. **Make sure that all information entered here is included in the Methods section of the manuscript.**<br><br>**Format for specific study types**<br><br>**Human Subject Research (involving human participants and/or tissue)**<br>• Give the name of the institutional review board or ethics committee that approved the study<br>• Include the approval number and/or a statement indicating approval of this research<br>• Indicate the form of consent obtained (written/oral) or the reason that consent was not obtained (e.g. the data were analyzed anonymously)<br><br>**Animal Research (involving vertebrate animals, embryos or tissues)**<br>• Provide the name of the Institutional Animal Care and Use Committee (IACUC) or other relevant ethics board that reviewed the study protocol, and indicate whether they approved this research or granted a formal waiver of ethical approval<br>• Include an approval number if one was obtained<br>• If the study involved *non-human primates*, add *additional details* about animal welfare and steps taken to ameliorate suffering<br>• If anesthesia, euthanasia, or any kind of animal sacrifice is part of the study, include briefly which substances and/or methods were applied<br><br>**Field Research**<br><br>Include the following details if this study involves the collection of plant, animal, or other materials from a natural setting:<br>• Field permit number<br>• Name of the institution or relevant body that granted permission	
**Data Availability**<br><br>Authors are required to make all data	Yes - all data are fully available without restriction

underlying the findings described fully available, without restriction, and from the time of publication. PLOS allows rare exceptions to address legal and ethical concerns. See the PLOS Data Policy and FAQ for detailed information.<br><br>A Data Availability Statement describing where the data can be found is required at submission. Your answers to this question constitute the Data Availability Statement and **will be published in the article**, if accepted.<br><br>**Important:** Stating 'data available on request from the author' is not sufficient. If your data are only available upon request, select 'No' for the first question and explain your exceptional situation in the text box.<br><br>Do the authors confirm that all data underlying the findings described in their manuscript are fully available without restriction?	
**Describe where the data may be found in full sentences. If you are copying our sample text, replace any instances of XXX with the appropriate details.**<br><br>• If the data are **held or will be held in a public repository**, include URLs, accession numbers or DOIs. If this information will only be available after acceptance, indicate this by ticking the box below. For example: *All XXX files are available from the XXX database (accession number(s) XXX, XXX.*).<br>• If the data are all contained **within the manuscript and/or Supporting Information files**, enter the following: *All relevant data are within the manuscript and its Supporting Information files.*<br>• If neither of these applies but you are able to provide **details of access elsewhere**, with or without limitations, please do so. For example:<br><br>*Data cannot be shared publicly because of [XXX]. Data are available from the*	Research data may be shared by the corresponding author upon request from other researchers.

XXX Institutional Data Access / Ethics Committee (contact via XXX) for researchers who meet the criteria for access to confidential data.<br><br>*The data underlying the results presented in the study are available from (include the name of the third party and contact information or URL).*<br>• This text is appropriate if the data are owned by a third party and authors do not have permission to share the data.<br><br>* typeset	
Additional data availability information:	

Manuscript
Click here to access/download;Manuscript;AK_Isokinetic Study_Main Manuscript.docx

Comparative Analysis of Isokinetic Ratios Between Hamstrings and Quadriceps in Wrestlers: Resting State Versus Post-Fatigue Evaluation

Ali Tatlıcı[1], Ahmet Kurtoğlu[2], Muhammed Yılmaz[3], Halil Güngör[4], Ömer Özer[5], Bekir Çar[6], Jarosław Muracki[7], Robert Trybulski[8], Monira I. Aldhahi[9]

[1] Department of Physical Education and Sport Teaching, Faculty of Sport Sciences, Selcuk University, 42060, Konya, Türkiye; alitatlici@selcuk.edu.tr

[2] Department of Coaching Education, Faculty of Sport Science, Bandirma Onyedi Eylul University, 10200, Balıkesir, Türkiye; kurtogluahmet18@gmail.com

[3] Department of Coaching Education, Faculty of Sport Sciences, Selcuk University, 42060, Konya, Türkiye; muhammed_04@icloud.com

[4] Department of Physical Education and Sport Teaching, Faculty of Sport Sciences, Selcuk University, 42060, Konya, Türkiye; halilgungor88@gmail.com

[5] Department of Physical Education and Sport Teaching, Faculty of Sport Sciences, Bandırma Onyedi Eylül University; 10200, Balıkesir, Türkiye; besyo4307@gmail.com

[6] Department of Physical Education and Sport Teaching, Faculty of Sport Sciences, Bandırma Onyedi Eylül University; 10200, Balıkesir, Türkiye; carbekir@gmail.com

[7] Institute of Physical Culture Sciences, Department of Physical Culture and Health, University of Szczecin, 70453 Szczecin, Poland; jaroslaw.muracki@usz.edu.pl

[8] Medical Department, Wojciech Korfanty Upper Silesian Academy, 40659 Katowice, Poland; rtrybulski.provita@gmail.com

[9] Department of Rehabilitation Sciences, College of Health and Rehabilitation Sciences, Princess Nourah bint Abdulrahman University, P.O. Box 84428, Riyadh 11671, Saudi Arabia. mialdhahi@pnu.edu.sa

*Correspondence: Ahmet Kurtoğlu (kurtogluahmet18@gmail.com), Monira I. Aldhahi (mialdhahi@pnu.edu.sa)

Abstract

Introduction: [illegible]s study aimed to determine whether the conventional hamstring-quadriceps ratio (H:Q_{Conv}) and functional hamstring-quadriceps ratio (H:Q_{Func}) are more indicative in resting and post-fatigue states, examine the H:Q_{Conv} and H:Q_{Func} isokinetic strength ratios, and analyze the contributions of hamstring and quadriceps strength changes to these ratios.

Materials and methods: The study involved 16 wrestlers who achieved rankings in national championships. Their average age was 1[illegible] ± 1.2 years, with an average height of 171.6 ± 8.6 cm and weight of 65.1 ± 16.6 kg. Their BMI was 21.8 ± 4.0 kg/m², and they had an average of 4.3 ± 1.0 years of training experience. The athletes participated in isokinetic assessments both at rest and after fatigue. The concentric and eccentric strengths of the hamstrings and concentric strength of the quadriceps were measured at angular velocities of 60°/s and 180°/s, respectively.

Results: Two-factor repeated-measures ANOVA revealed a highly significant main effect of time on Q_{CON} ($F_{(1,15)}=106.789$, $p<.001$, $\eta^2_p=.877$), and angular velocity and interaction were not significant. For H_{CON}, there was no time effect, but the velocity effect was significant (60°/s > 180°/s; $F_{(1,15)}=31.771$, $p<.001$, $\eta^2_p=.679$). For H_{ECC}, the time and interaction effects were insignificant, and the velocity remained at the threshold ($F_{(1,15)}=3.654$, $p=.075$). The H/Q_{conv} ratio generally increased after fatigue ($F=17.492$, $p=.001$), and there was a level difference between velocities ($F=9.147$, $p=.009$), but the interaction was insignificant. Both time ($F=59.489$, $p<.001$) and velocity ($F=6.716$, $p=.020$) were significant for the H/Q_{func} ratio, as was the time × velocity interaction ($F=4.708$, $p=.046$); the increase was particularly reliable at 60°/s ($\Delta=+0.131$, 95% CI [0.044, 0.218], $p=.006$). In the component decompositions, the increase in the time effect was shared similarly by the hamstrings and quadriceps, whereas the hamstrings' contribution significantly drove the angular velocity difference.

Conclusion: Fatigue significantly reduced the concentric torque of the quadriceps, whereas the concentric/eccentric outputs of the hamstrings were relatively preserved; consequently, the H/Q balance increased, particularly at 60°/s. The time effect was strong for functional H/Q, and a time × velocity interaction was present: the increase was reliable at 60°/s but not statistically confirmed at 180°/s. Component decomposition showed that the time-related change was shared to a similar extent by both muscles, whereas the angular velocity difference was predominantly explained by the hamstring contribution.

Keywords: Isokinetic Strength, H:Q Ratio, Muscle Fatigue, Wrestling.

Introduction

Wrestling is regarded as one of the most demanding Olympic events, consisting of repeated short-term high-intensity efforts [1]. This highly competitive environment has physiological consequences, such as lactate accumulation in muscles, disturbance of acid-base balance, and strength losses that primarily affect power in the lower extremity muscle groups [2]. Fatigue during sports activities decreases the potential for force production by lower extremity muscles [3], adversely influences isokinetic performance, and predisposes athletes to strength imbalances around the knee joint [4]. Excessive muscle fatigue can impair the H/Q balance and neuromuscular control, thereby reducing knee stability. This condition increases the risk of valgus angulation with sudden "buckling" reactions during dynamic movements, such as landing, changing direction, or making a move, thus contributing to the risk of injury [5]. Knee injuries are among the most common injuries in wrestling and have been reported to account for 26.1% of injuries among wrestlers in the United Kingdom [6]. Furthermore, it has been reported that competition injury rates are approximately 40 times higher than training injury rates, with knee injuries capable of keeping an athlete out of competition for weeks [7].

It has been stated that maintaining the strength balance between the hamstring and quadriceps muscles (H:Q ratio) might protect against hamstring strains and anterior cruciate ligament injuries [8]. The H:Q ratio, which is crucial for maintaining knee joint stability, is often used as a measure of the risk of lower extremity injuries [9, 10]. The $H:Q_{Conv}$ ratio is approximately 0.66 [10]. The functional $H:Q_{Func}$ ratio is usually 1.0 or more [11] and indicates the relationship between H_{ECC} and Q_{CON}; this may be more realistic for measuring sport-specific movements [12]. These H:Q ratios have become common in professional teams as part of performance assessments and injury risk evaluations [3]. In wrestling, the H:Q ratio is an essential parameter for assessing performance, balance in muscle strength, and injury risk [13, 14].

A review of the literature reveals that most strength assessments are performed under resting conditions [15]. Özbay and Ulupınar [16] noted the limitation of this approach by reporting that resting tests could not distinguish performance differences, while measurements taken after fatigue clearly revealed distinctions between elite and super-elite wrestlers. Similarly, Machado, Nakamura [3] and Pinto, Blazevich [4] emphasized that evaluating parameters such as the H:Q ratio only under resting conditions does not accurately reflect the injury risk. In fact, Liu, Peng [17] reported that fatigue might have a significant negative effect on muscle strength and motor control leading to imbalances in the knee joint which increases risks for ACL and hamstring injuries. It has

also been shown that in isokinetic fatigue tests hamstring muscles are more adversely affected than quadriceps muscles [18]. Other studies have similarly demonstrated a significant decrease in the H:Q ratio with increasing fatigue levels [19]. The tournament format of wrestling requires athletes to compete in up to five matches within one day, with very short recovery intervals between these matches, resulting in the rapid accumulation of muscle fatigue [20]. Increasing fatigue therefore causes disproportionate strength loss between the quadriceps and hamstring muscles, disrupting the H:Q ratio, reducing stability at the knee joint, and increasing the risk of injury [3, 4]. Assessments conducted under fatigue conditions in wrestling can provide more realistic sport-specific insights into an athlete's capacity for muscle strength, H:Q ratio, and endurance profiles of the hamstring and quadriceps beyond what can be obtained from resting tests. Therefore, the present study aimed to examine the profiles of muscle strength around the knee joint among Greco-Roman wrestlers by evaluating the ratios of $H:Q_{Conv}$ and $H:Q_{Func}$ under both resting and post-fatigue conditions, in addition to determining the relationships between these parameters and injury risk.

Materials and Methods

Study design and participants

A crossover design was employed involving 16 male Greco-Roman wrestlers from the Konya Athlete Training Center (SEM), all of whom were actively engaged in regular wrestling training and voluntarily participated in the study as national-level competitors. The athletes were classified according to the McKay Participant Classification Framework [21] as Tier 3 Highly Trained National Level athletes given their participation. The participants' mean age was [illegible] ± 1.21 years, height 171.6 ± 8.6 cm, body weight 65.1 ± 16.6 kg, and body mass index (BMI) 21.8 ± 4.0 kg/m². Their average training experience was [illegible] ± 1.0 years, and they were actively engaged in regular Greco-Roman wrestling training programs. The wrestlers were in the preparation phase before the league season began. The athletes were instructed to avoid any meal (except hydration with water) for at least one hour before the assessments. None of the participants had experienced any musculoskeletal injury or condition affecting lower limb function for at least three months before the study. All wrestlers were familiar with the isokinetic knee extension and flexion tests, as they had performed similar assessments in previous seasons. All athletes completed the isokinetic evaluations, and no adverse events were reported during the testing. All participants and their legal guardians were informed about the study procedures, and written informed consent was obtained prior to their participation. The study protocol was approved by the Selçuk University Faculty of Sports Sciences Ethics Committee (protocol number 92, 16 July 2024, Ethics Committee of Selcuk University, Faculty of Sports Science, Konya, Turkey) and was conducted in accordance with the principles of the Declaration of Helsinki.

Procedure

This study was based on an experimental design and was conducted over three separate testing days. On the 1st visit, the participants were familiarized with the study procedures to ensure proper adaptation to the measurement protocol (no strength record). On the 2nd visit, C[illegible]ex isokinetic strength tests were performed by athletes under rest conditions, and these measurements were recorded as rest values. On the 3rd day, an exhaustive exercise protocol was applied to participants, who then completed another round of Cybex isokinetic tests immediately after (within 30 s). The measurements were recorded as fatigue values. All testing sessions took place between 09:00 and 11:00 a.m., under standardized [illegible]boratory conditions, with a 48-hour rest period between each testing day to allow for full recovery. Thus, all measurements for each athlete were obtained on three separate days. This design permitted comparisons of muscle strength profiles and H:Q ratios under both rest and fatigue conditions, which are more representative of actual wrestling-specific fatigue, thus providing more practical insights into [illegible]formance and injury risk.

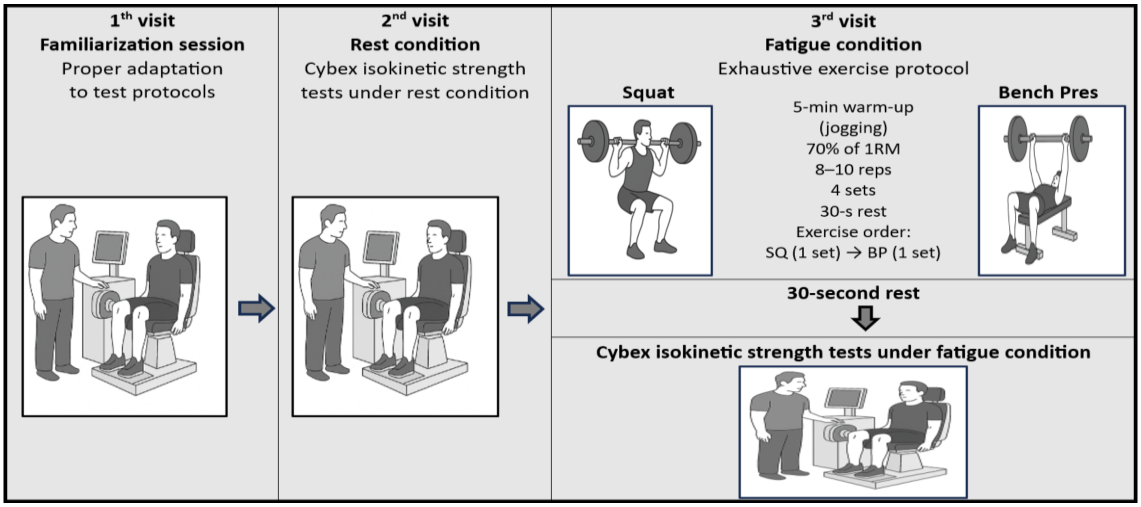

Figure 1. Study design

Fatigue-Inducing Exercise Protocol

All subjects were standardized to a similar level of fatigue using an exercise protocol. A simulated wrestling match was avoided for this purpose, given that any variation between wrestlers in their individual tactics and techniques, whether attacking, defending, or applying different holds, could result in unequal levels of fatigue. Therefore, squat (SQ) and bench press (BP), which involve large muscle groups in the lower and upper body, respectively, were selected to predominantly activate the anaerobic energy systems that are heavily utilized in wrestling bouts. Each subject completed four sets at a load equivalent to

70% of their one-repetition maximum (1RM), with 8-10 repetitions for both SQ and BP. The exercises alternated between each other, with one set of SQ followed immediately by one set of BP. Passive rest was allowed for 30 s between all sets and exercises performed under this protocol. The athletes completed a 5-minute jog warm-up prior to the start of the protocol and were verbally encouraged during the exercise period to achieve maximum effort [16].

Isokinetic Strength Measurement

The strength of the quadriceps and hamstring muscles was determined using a Cybex isokinetic dynamometer (Cybex NORM®, Humac, CA, USA, 2004) in the Kinanthropometry Laboratory at Selçuk University Sports Science Faculty. Participants performed a standardized five-minute warm-up on a cycle ergometer prior to testing at a pedaling cadence of 60–70 revolutions per minute to prepare for the isokinetic evaluation. All participants underwent familiarization consisting of three submaximal repetitions at angular velocities of 6[illegible]s and 180°/s in both concentric and eccentric modes before the actual testing session to ensure adaptation to the protocol [22]. The order of the angular velocity and contraction mode testing was randomized for each participant using a Latin square design. During measurement, subjects were properly positioned on the dynamometer chair with straps across the thigh and trunk secured against any extraneous movement, also instructed to hold lateral handles of chair for stabilization duri[illegible]ests [23]. The lever arm length was individually adjusted to the resistance pad just proximal to the medial malleolus, and contralateral leg was hanging freely without an external load. The axis of the knee joint was aligned with the rotation axis of the dynamometer lever arm at the distal end of the lateral femoral condyle [11]. Concentric muscle torque for quadriceps and hamstrings was measured by three maximal contractions in concentric-concentric mode at 60°/s over a range from 90° to 10° knee flexion similarly assessed eccentric torque with three maximal contractions in eccentric-eccentric mode moving from 10° to 90°. The same protocol was repeated at an angular velocity of 180°/s. A passive rest interval of two minutes between test conditions minimized fatigue (Magris et al[illegible]2024). Standardized verbal encouragement was provided throughout the procedure to achieve maximal effort, along with real-time visual feedback displayed on a computer screen. For each contraction type, the peak torque value was recorded and used for statistical analysis; the highest obtained value was considered [23].

Statistical Analyses

All analyses were conducted using R (v4.x). First, a 2×2 repeated measures ANOVA was applied for the variables (Q_{CON}, H_{CON}, H_{ECC}, H/Q_{Conv}, H/Q_{Func}) to test the main effects of time (rest–fatigue) and angular velocities (60°/s–180°/s) and the time ×Angular Velocity

interaction. ANOVA results were reported using F, p, and partial eta-squared (η^2 p). The magnitude of the effect sizes was interpreted as follows: η^2p = 0.01 (small), η^2p = 0.06 (medium), and η^2p = 0.14 (large) [24]. Assumptions were checked at the data/residual level: normality (Shapiro–Wilk), outlier checks (±3 SD), and effect diagnostics. The significance threshold was set at α=0.05, and p-values for multiple pairwise comparisons were corrected using the [illegible]m method.

Figures (raincloud) show raw data points, density (half-violin), mean, and 95% confidence interval (CI) for each condition. To show simple effects, matched comparisons by angular velocity (post–rest; 60–180) were performed, and the mean difference (Δ), 95% confidence interval, p, and matched Cohen's d (d^z) were calculated. Log-ratio decomposition was applied to examine which component drove the H/Q ratios: for H/Q_{conv}, $\Delta\log(H_{KON})$ and $-\Delta\log(Q_{KON})$; for H/Q_{func}, $\Delta\log(H_{ECC})$ and $-\Delta\log(Q_{KON})$ were calculated. For the contrasts of time (resting → fatigue) and angular velocities (60→180), within-subject contributions were obtained, and the difference in the contribution of the quadriceps and hamstrings was tested using a paired t-test and reported with d^z and 95% CI. The packages ggplot2/ggdist were used for visualization. All tests were two-tailed, and summaries were presented as mean ± SD (or Δ for ratios/contributions, 95% CI).

Results

Table 1 presents the mean and standard deviation values of the quadriceps and hamstring torques, as well as the $H{:}Q_{Conv}$ and $H{:}Q_{Func}$ ratios, measured at 60°/s and 180°/s under pre- and post-fatigue conditions.

Table 1. Descriptive statistics of quadriceps and hamstring torque values and hamstring-to-quadriceps (H:Q) ratios at 60°/s and 180°/s under rest and fatigue conditions.

Angular velocity (°/s)	Time	Q_{CON} (Nm)	H_{CON} (Nm)	H_{ECC} (Nm)	$H{:}Q_{Conv}$	$H{:}Q_{Func}$
60°/s	Rest	131.75± 41.78	101.56± 33.43	110.56 ± 35.34	0.77 ± 0.06	0.84 ± 0.07
	Fatigue	122.13± 32.10	105.81± 29.43	116.56 ± 29.11	0.87 ± 0.15	0.97 ± 0.17
180°/s	Rest	95.69 ± 31.80	84.50 ± 25.23	108.56 ± 29.71	0.90 ± 0.13	1.17 ± 0.20
	Fatigue	92.69 ± 26.75	86.69 ± 24.41	109.06 ± 26.33	0.94 ± 0.11	1.20 ± 0.18

Table 2 presents the results of the two-factor repeated-measures ANOVA for Time (pre–post) × Velocity (60°/s–180°/s). The main effect of time for Q_{CON} was very large ($F_{(1,15)}$=106.789, p<.001, η^2_p=.877), whereas the main effect of velocity ($F_{(1,15)}$ =2.034, p=.174, η^2_p=.119) and the interaction ($F_{(1,15)}$=2.545, p=.132, η^2_p=.145) were not significant. There was no time effect in H_{CON} ($F_{(1,15)}$=0.833, p=.376, η^2_p=.053), but the main effect of angular velocity was significant ($F_{(1,15)}$=31.771, p<.001, η^2_p=.679); and the interaction was insignificant ($F_{(1,15)}$=0.458, p=.509, η^2_p=.030). Neither time (F(1,15)=0.751, p=.400, η^2_p=.048) nor interaction ($F_{(1,15)}$=2.604, p=.127, η^2_p=.148) were significant for H_{ECC}; the angular velocity effect was at the trend level ($F_{(1,15)}$=3.654, p=.075, η^2_p=.196). Both the

main effects of time ($F_{(1,15)}$=17.492, p=.001, η^2_p=.538) and angular velocity ($F_{(1,15)}$=9.147, p=.009, η^2_p=.379) were significant for the H/Q_{Conv} ratio, but the interaction was not significant ($F_{(1,15)}$=2.079, p=.170, η^2_p=.122). The main effects of time ($F_{(1,15)}$=59.489, p<.001, η^2_p=.799) and velocity ($F_{(1,15)}$=6.716, p=.020, η^2_p=.309) on the H/Q_{Func} ratio, as well as the Time × Velocity interaction, were significant ($F_{(1,15)}$=4.708, p=.046, η^2_p=.239).

Table 2. Results of the 2×2 repeated-measures ANOVA of quadriceps and hamstring strength

Parameter	Effect	F(1,15)	p	η^2_p
**Q_{CON}** (Nm)	Time	106.789	< .001***	.877
	Velocity	2.034	.174	.119
	Time × Velocity	2.545	.132	.145
**H_{CON}** (Nm)	Time	0.833	.376	.053
	Velocity	31.771	< .001***	.679
	Time × Velocity	0.458	.509	.030
**H_{ECC}** (Nm)	Time	0.751	.400	.048
	Velocity	3.654	.075	.196
	Time × Velocity	2.604	.127	.148
**H/Q_{Conv}**	Time	17.492	.001**	.538
	Velocity	9.147	.009**	.379
	Time × Velocity	2.079	.170	.122
**H/Q_{Func}**	Time	59.489	< .001***	.799
	Velocity	6.716	.020*	.309
	Time × Velocity	4.708	.046*	.239

Note. Velocity: angular velocity, the asterisk (*) indicates a statistically significant difference (*p < .05, **p < .01, **p < .001).

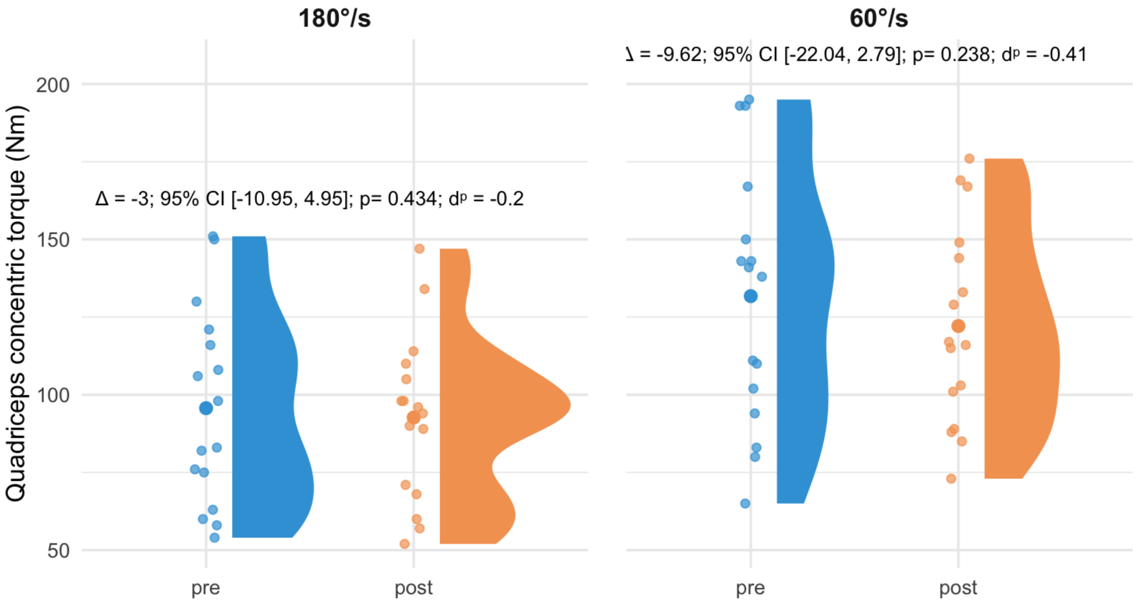

Figure 2. Raincloud plot of resting (pre) and post-fatigue values for the concentric torque of the quadriceps (Q_{CON}) at 60°/s and 180°/s: The points represent individual observations, the half-violin distribution represents the sample distribution, the horizontal lines represent group means, and the short lines above them represent the 95% confidence interval.

Figure 2 shows that post-fatigue concentric torque of the quadriceps tends to be lower than the pre-fatigue state for both 60°/s and 180°/s; however, the difference is not statistically significant as the confidence intervals include zero (60°/s: Δ=−3.00 Nm, 95% CI [−10.95, 4.95], p=.434, d=−0.20; 180°/s: Δ=−9.62 Nm, 95% CI [−22.04, 2.79], p=.238, d=−0.41). This pattern indicates a general decrease at both angular velocities, and the main effect of Time in the ANOVA test results was very large and significant, consistent with the results that the main effect of velocity and the Time×Angular Velocity interaction were not significant. That is, the reducing effect of fatigue on Q_{CON} appears to be independent of velocity; although the differences within each angular velocity in the figure do not individually cross the statistical threshold (because the CIs include zero), when all conditions are considered together, the omnibus time effect is strongly detected (particularly because Holm correction and smaller samples split by angular velocity may have prevented significance in simple effects). Practically, a similar decrease in the concentric capacity of the quadriceps muscle after fatigue should be expected at both angular velocities; velocity does not significantly moderate this decrease.

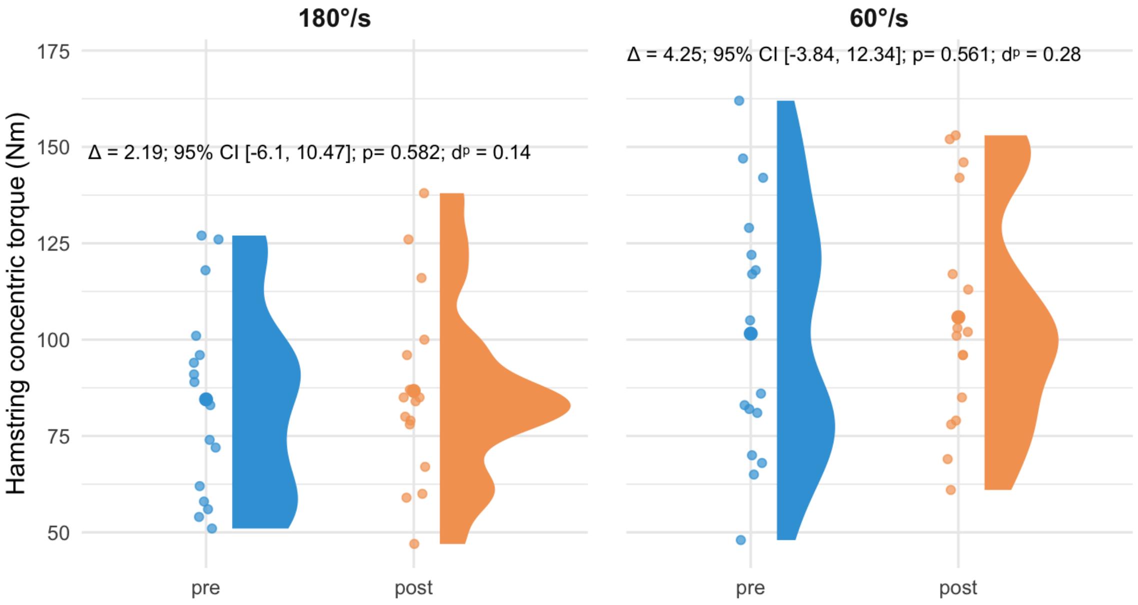

Figure 3. Raincloud plot of resting (pre) and post-fatigue values for the concentric torque of the hamstring (H_{CON}) at 60°/s and 180°/s: The points represent individual observations, the half-violin distribution represents the sample distribution, the horizontal lines represent group means, and the short lines above them represent the 95% confidence interval.

As shown in Figure 3, at 180°/s, fatigue showed a small and insignificant increase compared to the resting state (Δ=+2.19 Nm; 95% CI [−6.10, 10.47]; p=.582; d≈0.14). Similarly, there was a small and non-significant increase at 60°/s (Δ=+4.25 Nm; 95% CI [−3.84, 12.34]; p=.561; d≈0.28). These pairwise comparisons are consistent with the findings of the two-factor repeated-measures ANOVA: the main effect of time was not significant, and the time ×Angular Velocity interaction was also not significant; therefore, the effect of fatigue on H_{CON} remained similar and small at both angular velocities. In contrast, the main effect of angular velocity was strong, confirming the tendency for absolute torque levels at 60°/s to be significantly higher than those at 180°/s. The inclusion of zero in the confidence intervals and the insignificance of the time/interaction terms indicate that the H_{CON} changes after fatigue in this sample are clinically small and statistically uncertain. In practice, this suggests that hamstring concentric performance is angular velocity-dependent (60°/s > 180°/s) but relatively resistant to fatigue; therefore, while angular velocity differences should be considered in monitoring/recovery strategies, short-term H_{CON} changes post-fatigue alone should not be decisive.

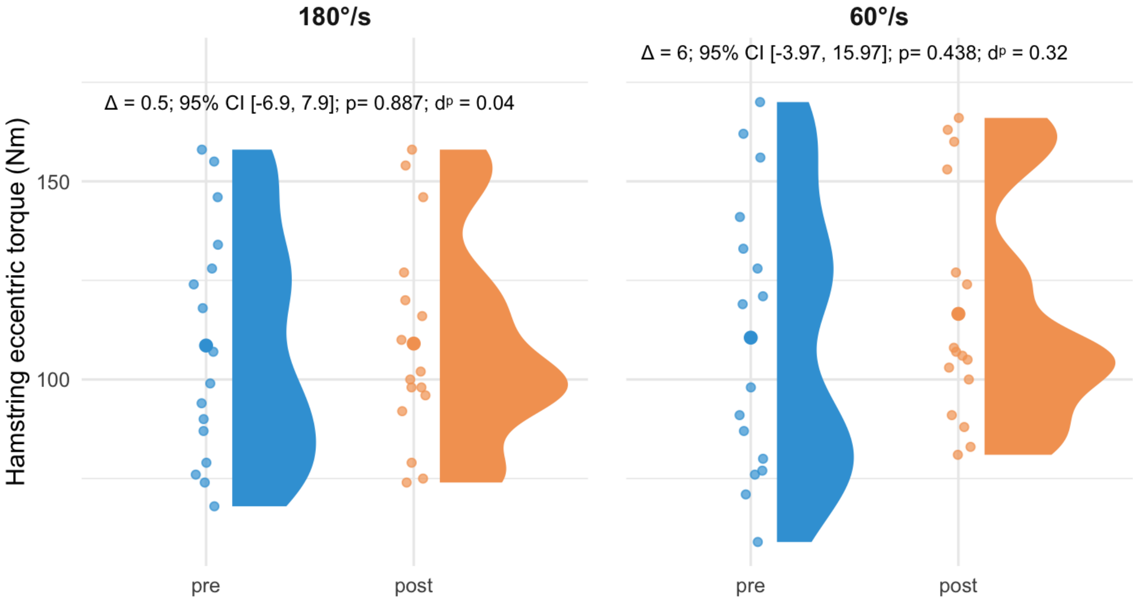

Figure 4. Raincloud plot of resting (pre) and post-fatigue values for the eccentric torque of the hamstring (H_{ECC}) at 60°/s and 180°/s: The points represent individual observations, the half-violin distribution represents the sample distribution, the horizontal lines represent group means, and the short lines above them represent the 95% confidence interval.

The two-factor repeated-measures ANOVA indicated that the main effect of time was not significant for H_{ECC}, the main effect of Angular Velocity remained at the statistical limit, and the Time×Angular Velocity interaction was not significant. The paired comparisons reported in Figure 4 are consistent with this table: at 180°/s, post-fatigue torque showed a very small and unreliable increase compared to the resting condition (Δ=+0.50 Nm; 95%

CI [−6.90, 7.90]; p=.887; d≈0.04); the increase at 60°/s appeared larger but was not statistically significant (Δ=+6.00 Nm; 95% CI [−3.97, 15.97]; p=.438; d≈0.32). The inclusion of zero in the confidence intervals and the lack of significance for both the time and interaction terms in the ANOVA suggest that hamstring eccentric performance is generally robust against the fatigue protocol; possible angular velocity differences did not reach the level of evidence in this study. In practical terms, a significant deterioration in eccentric hamstring torque should not be expected in post-fatigue testing; the small increase observed at 60°/s should be interpreted clinically while considering the statistical uncertainty.

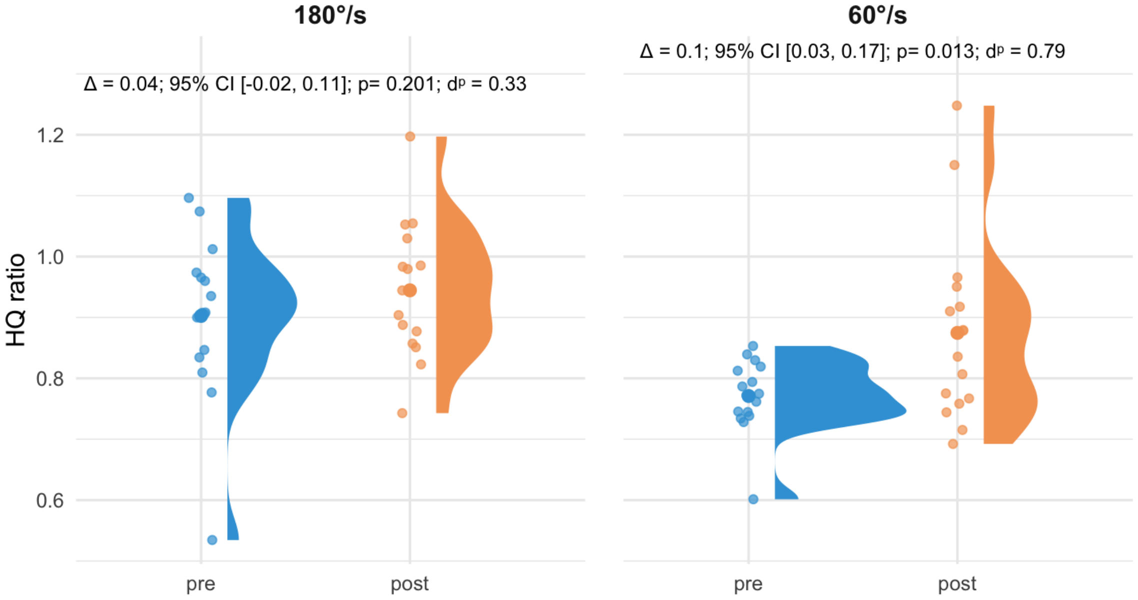

Figure 5. Raincloud plot of resting (pre) and post-fatigue values for the H/Q_{Conv} at 60°/s and 180°/s: The points represent individual observations, the half-violin distribution represents the sample distribution, the horizontal lines represent group means, and the short lines above them represent the 95% confidence interval.

Figure 5 shows that the post-fatigue ratio at 60°/s in the HQconv group exhibited a significant and relatively large increase compared to the resting state (Δ=+0.10; 95% CI [0.03, 0.17]; p=.013; d≈0.79). In contrast, the post-fatigue increase at 180°/s was small and statistically uncertain (Δ=+0.04; 95% CI [−0.02, 0.11]; p=.201; d≈0.33). The fact that the confidence interval at 60°/s does not include zero indicates a reliable improvement in the H/Qconv balance from rest to post-fatigue, whereas the inclusion of zero at 180°/s suggests that this difference cannot be demonstrated at the sample level at high angular velocity. This pattern of figures indicates that there is a general increase after fatigue (main effect of Time: F(1,15)=17.492, p=.001, η^2_p=.538) and a general difference in level between angular velocities (Velocity: $F_{(1,15)}$=9.147, p=.009, η^2_p=. 379); however, it supports that the increase

did not differ significantly according to velocity (Time×Velocity: $F_{(1,15)}$=2.079, p=.170, η^2_p=.122). In practice, this indicates that the post-fatigue conventional H/Q balance increases in a clinically significant manner compared to the resting state, particularly at a low angular velocity (60°/s), while the change remains small and uncertain at a high angular velocity (180°/s). This indicates that while the general trend of balance improvement from rest to post-fatigue remains independent of angular velocity, its magnitude is more apparent at 60°/s. In this context, an increase in the H/Q_{conv} ratio reflects a change in the relative contributions of the hamstrings and quadriceps; therefore, "improvement" mostly results from Q_{CON} (the denominator) decreasing more with fatigue, while H_{CON} (the numerator) is less affected/relatively preserved. In other words, an increase in the ratio does not necessarily mean that the hamstrings have increased in absolute terms; in most cases, the decrease in the quadriceps is greater than that in the hamstrings. Therefore, the relative balance shifts in favor of hamstrings. This pattern demonstrates that the direction of the balance shift during the transition from rest to fatigue is determined by the differing sensitivities of the two muscle groups, with the increase essentially reflecting the differential fatigue effect.

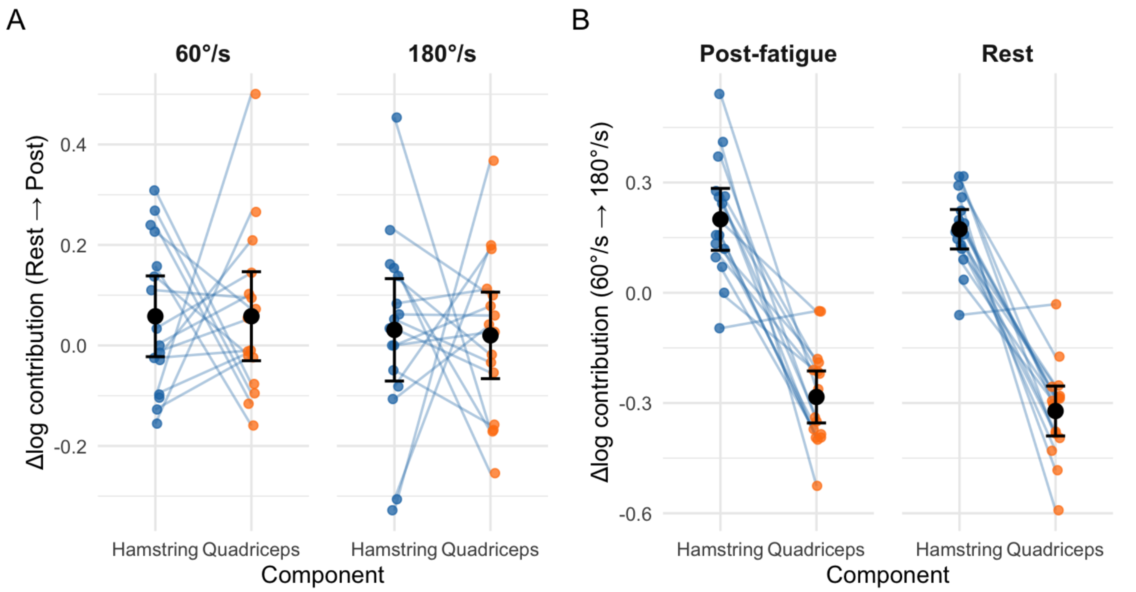

Figure 6. Separation of component contributions in the H/Q_{conv} ratio on a log scale: **Panel 6A:** Separated representation of the H/Qconv change from rest to post-fatigue (Rest → Post) into hamstring (ΔlogH) and quadriceps (−ΔlogQ) contributions at each angular veloH/QConv°/s, 180°/s). **Panel 6B:** Effect of angular velocity difhamstring60°/s → 180°/quadricepsonv, decomposed into Hamstring and Quadriceps contributions for each time level (Rest, Post-fatigue).

Figure 6A shows the distribution of contributions to the change from rest to post-fatigue according to angular velocities, and the contributions of the hamstrings (ΔlogH) and quadriceps (−ΔlogQ) appear to be of equal magnitude at both angular velocities; therefore, there is no evidence that a single muscle predominantly drives the increase in H/Qconv

observed at 60°/s post-fatigue (60°/s: hamstring − quadriceps = 0.000, 95% CI [−0.151, 0.151]; $p = .999$; $d^z = 0.00$; 180°/s: 0.011, [−0.162, 0.184]; $p = .894$; $d^z = 0.03$).

Figure 6B shows which component accounts for the difference in angular velocity between 60°/s and 180°/s, separately for at rest and after fatigue; here, the hamstring contribution is significantly higher, and the differences are consistently above zero with wide confidence intervals (Post-fatigue: Hamstring − quadriceps = 0.483, 95% CI [0.353, 0.613]; $p< .001$; $d^z = 1.98$. Resting: 0.494, [0.389, 0.600]; $p < .001$; $d^z = 2.50$). This pattern indicates that both muscles contribute similarly to the time effect, but the hamstrings are dominant in the angular velocity effect.

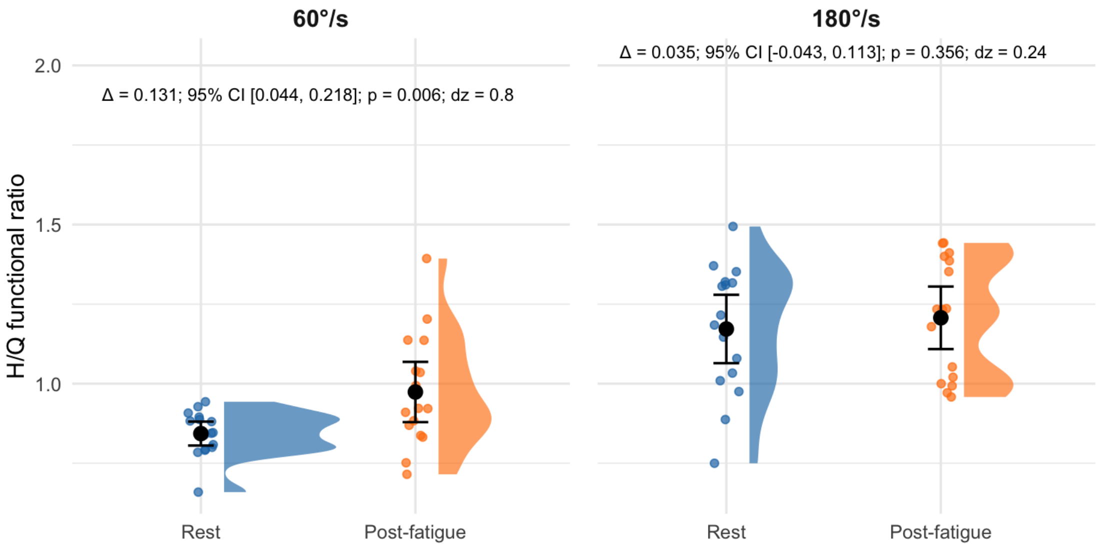

Figure 7. Raincloud plot of resting (pre) and post-fatigue values for the H/Q_{func} at 60°/s and 180°/s: The points represent individual observations, the half-violin distribution represents the sample distribution, the horizontal lines represent group means, and the short lines above them represent the 95% confidence interval.

Figure 7 shows that the functional H/Q ratio increased significantly at 60°/s compared to the resting state after fatigue; however, the change remained small and unreliable at 180°/s (60°/s: Δ=+0.131; 95% CI [0.044, 0.218]; $p=0.006$; $d^z=0.80$; 180°/s: Δ=+0.035; 95% CI [−0.043, 0.113]; $p=0.356$; $d^z=0.24$). This figure pattern is consistent with the two-factor repeated measures ANOVA results: the main effect of time is strong and generally post-fatigue > rest, and there is also a general level difference between angular velocities. Importantly, the Time×Angular Velocity interaction was significant. In this case, the enhancing effect of fatigue was evident and reliable at 60°/s but could not be statistically confirmed at 180°/s (the confidence interval included zero). Therefore, it can be said that

the increase in functional H/Q balance arises from the effect of time; however, its magnitude varies with angular velocity and is particularly visible at 60°/s.

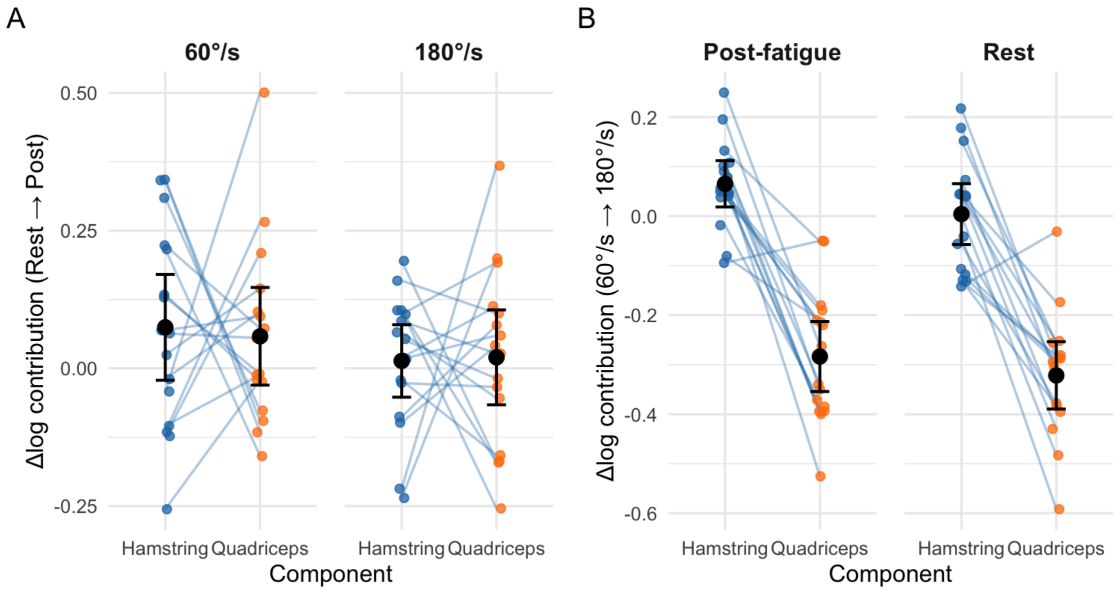

Figure 8. Separation of component contributions in the H/Q_{Conv} ratio on a log scale: **Panel 8A:** Separated representation of the $H/Q_{,Conv}$ change from rest to post-fatigue (Rest → Post) into Hamstring (ΔlogH) and Quadriceps (−ΔlogQ) contributions at each angular velocity (60°/s, 180°/s). **Panel 8B:** Effect of angular velocity difference (60° /s → 180° /s) on H/Q_{Conv}, decomposed into Hamstring and Quadriceps contributions for each time level (Rest, Post-fatigue).

In Panel 8A, in the transition from rest to post-fatigue, the contributions of the hamstrings (ΔlogH) and quadriceps (−ΔlogQ) appear to be similar in magnitude at both angular velocities; that is, there is no evidence that a single muscle 'dominantly' drives the increase in balance observed at 60°/s post-fatigue (60°/s: Δ=+0.016, 95% CI [−0.146, 0.179]; p=0.833; dz=0.05; 180°/s:Δ=−0.007, [−0.143, 0.130]; p=0.919; dz=−0.03). This pattern indicates that the relative contributions of the hamstrings and quadriceps remain balanced.

Panel 8B shows the component responsible for the angular velocity difference between 60°/s and 180°/s separately for rested and post-fatigue conditions. The hamstring contribution was significantly higher at both time points, and the differences were consistently above zero (post-fatigue: Δ=+0.349, 95% CI [0.254, 0.444]; p<.001; dz=1.96; Rest: Δ=+0.326, [0.232, 0.420]; p<.001; dz=1.85). This pattern indicates that the hamstrings are dominant in the angular velocity effect; in contrast, for the time effect, both muscles contribute to a similar extent.

Discussion

The present study examined changes in knee joint muscle strength and the $H:Q_{Conv}$ and $H:Q_{Func}$ ratios in Greco-Roman wrestlers pre- and post-fatigue. Both ratios increased following fatigue, primarily due to a significant decrease in quadriceps strength, whereas hamstring strength remained relatively unchanged. Despite both ratios increasing post-fatigue, the magnitude of change was velocity-dependent, with a significant increase in the $H:Q_{Func}$ ratio at 60°/s but not at 180°/s. Additionally, post-hoc analysis indicated that fatigue affected both muscle groups similarly at both angular velocities, as evidenced by the absence of interaction effects, suggesting that the effects of fatigue were additive. The apparent difference between the angular velocities was attributed to a greater percentage contribution from the hamstrings at 60°/s compared to 180°/s.

Wrestling consists of numerous short, high-intensity efforts performed intermittently within a match or across tournament days [25]. During tournaments, athletes typically perform multiple bouts in a single day, which may result in significant performance decrements [20]. K[illegible]emer, Fry [2] also described substantial reductions in the strength and explosive power of wrestlers between successive matches on the same day. Fatigue has been associated with impaired muscle function and an increased risk of hamstring strain [[illegible] and anterior cruciate ligament injuries [27]. Although no study has directly addressed this issue in wrestling, Wright, Ball and Wood [28] reported increases in both conventional and functional H:Q ratios following fatigue in soccer players ($H:Q_{Conv}$: pre = 0.75, post = 1.02; H:QFunc: pre = 0.88, post = 1.08). They attributed these changes to greater hamstring coactivation during quadriceps contractions under fatigue conditions, indicating that the hamstrings acted as stabilizers to protect the knee joint W[illegible]ght, Ball and Wood [28]. Thus, there could be an even greater performance loss for the quadriceps due to its own fatigue and increased antagonist resistance from the hamstrings. In this study, a similar phenomenon seems to explain the increase in the $H:Q_{Conv}$ and $H:Q_{Func}$ ratios after fatigue events.

After fatigue, both the $H:Q_{Conv}$ and $H:Q_{Func}$ ratios increased, mainly due to a significant decrease in quadriceps strength, while the hamstrings remained relatively unchanged. This indicates that fatigue had a greater effect on the quadriceps, thereby shifting the balance between the two muscle groups. The logarithmic contribution analysis supported this finding, as it showed almost equal contributions of the quadriceps and hamstrings to the ratio changes with fatigue. These results are directly related to the effect of the fatigue protocol on each of these specific muscle groups. Wrestling actions, such as pushing,

lifting, and maintaining an upright combat position, require repetitive activation of the quadriceps [1, 29]. The current study protocol, consisting of repeated sets of squats and bench presses at 70% 1RM with short rest periods, primarily stressed the quadriceps and likely led to marked strength reduction in that area while having lesser effects on hamstring strength. This difference in muscle involvement may also explain why some studies have found opposite results. For instance, in soccer, futsal, basketball, and handball, fatigue affects the hamstrings more severely, thereby decreasing the H:Q ratio [3, 4, 18, 30-34]. This is because they are highly engaged during sprinting and kicking motions, as well as rapid changes in direction, which involve intense eccentric muscle contractions. In addition to having a higher percentage of type II fibers [35], complex muscle architecture, and being biarticular, these characteristics make the hamstrings more vulnerable to fatigue [32]. These physiological characteristics may also help explain why, in the present study, the hamstrings played a leading role in the velocity-related changes in the H:Q ratios identified through logarithmic contribution analysis. Furthermore, this study is among the very few that have applied a logarithmic contribution analysis to examine muscle-specific determinants of H:Q ratio changes under different fatigue and angular velocity conditions.

The exercise protocol that causes fatigue used in the present study (squat and bench press at 70% of 1RM with short rest intervals) was previously reported by Özbay and Ulupınar [16] as a method to create wrestling-specific fatigue and has been validated in the literature. It was designed to simultaneously activate major upper- and lower-body muscle groups, thereby engaging anaerobic energy systems similar to those used during wrestling bouts. A simulated match was avoided because individual strategies and techniques among wrestlers, such as attacking, defending, and grappling, could lead to inconsistent levels of fatigue. Instead, a standardized approach ensured that all the participants experienced an equivalent degree of fatigue. Post-fatigue protocol testing revealed a significant decrease in quadriceps strength, with no statistically significant change in hamstrings, an expected outcome based on the nature of the protocol, which primarily involved efforts demanding quadriceps support relative to technical and physiological demands in wrestling. Furthermore, this reduction in performance cannot be attributed purely to peripheral fatigue, as protective mechanisms within the central nervous system can reduce motor neural drive, contributing to additional strength loss during repeated high-intensity efforts [32, 36]. This may further explain the decline in quadriceps strength with an increase in the H:Q ratio. In this sense, one could consider the applied protocol as a valid and reliable means for inducing wrestling-specific fatigue in suitable model experimental research within the field.

[illegible]his study, both the $H:Q_{Conv}$ and $H:Q_{Func}$ ratios increased after fatigue. However, only the functional ratio had a time×velocity interaction that was significantly different, indicating that it is more sensitive to changes in both fatigue and contraction angular velocity. The effect sizes confirmed this, with larger effects for the functional ratio, while

smaller changes were observed for the conventional ratio. This is consistent with previous studies that found the H:Q$_{Func}$ ratio to be a better measure of neuromuscular balance and injury risk [4]. Croisier, Ganteaume [8] found that reduced H:Q$_{Func}$ ratios are one of the most common imbalances in athletes and are a better predictor of injury risk than reduced H:Q$_{Conv}$ ratios. Baroni, Ruas [37] also stated that the H:Q$_{Func}$ ratio is more reflective of muscle coordination demands specific to sports, particularly at high angular velocities. These results highlight the importance of assessing the H:Q$_{Func}$ ratio, especially under conditions of fatigue and different angular velocities, as a more informative and practical approach for monitoring muscular balance and injury susceptibility in athletes.

This study has several limitations. First, because of the relatively small sample size, these findings cannot be generalized to the g[illegible]ter wrestling community or athletes from other sports. Although the fatigue protocol was carefully designed to replicate the metabolic and muscular demands specific to wrestling, it may not have fully captured the technical variability and psychological stress occurring in real matches. In addition, a cross-sectional design cannot establish a direct causal link between post-fatigue changes seen in the H:Q$_{Conv}$ and H:Q$_{Func}$ ratios with the actual risk of injury. Thus, future studies should include larger cohorts with simulated match conditions using longitudinal designs for better verification and expansion of the current results. Despite these limitations, this study takes an important step toward understanding post-fatigue muscle function among wrestlers and gaining insight into the sport-specific mechanisms of fatigue within this population.

Conclusion

This study examined the effects of a fatigue-inducing protocol on the muscle strength profiles of Greco-Roman wrestlers. They found a significant decrease in quadriceps strength after fatigue, whereas the reduction in hamstring performance was relatively small. Consequently, both the H:Q$_{Conv}$ and H:Q$_{Func}$ ratios increased after fatigue. Of these two ratios, the H:Q$_{Func}$ was more sensitive to changes in angular velocity and therefore might be better for capturing high-angular velocity, sport-specific demands in wrestling. In short, the technical and physiological characteristics of wrestling seem to lead to greater fatigue-related declines in the quadriceps, contributing to the observed increases in the H:Q ratios. These results suggest that, particularly at the lower angular velocity of 60 °/s, the HQ$_{Func}$ ratio demonstrated a significant increase and may therefore be a more sensitive measure of fatigue muscle balance as it relates to potential injury risks for wrestlers. Thus, both fatigue and movement velocity should be considered when developing training strategies and injury prevention programs for athletes in this group.

Declarations

Consent for publication: Not applicable

Conflicts of Interest: The authors declare no conflicts of interest. The funders had no role in the study design, data collection, data analysis, data interpretation, manuscript writing, or decision to publish the results.

Data availability statement: The data presented in this study are available upon request from the corresponding author of this paper. The data are not publicly available for further publication because of restrictions related to the ongoing work.

Ethics approval and consent to participate: All procedures and research protocols in this study involving human participants were approved by the ethical standards of the Institutional Review Board (protocol number 92, 16 July 2024, Ethics Committee of Selcuk University, Faculty of Sports Science, Konya, Turkey). Written Informed consent was obtained from each participant, which was a condition for enrolment in the study, in accordance with the Declaration of Helsinki.

Funding: The authors received no specific funding for this work.

Author Contributions: Conceptualization and Methodology, A.T; A.K; M.Y; H.G;O.O; N.C; J.M; R.T and M.I.A; Data curation: A.T; A.K; M.Y; H.G;O.O; N.C; J.M; and R.T Writing —original draft preparation: all authors. Writing, review, and editing; all authors. All authors have read and agreed to the published version of this manuscript.

Acknowledgements: We extend our gratitude to the participants for their invaluable contribution to this study. Special thanks to the Princess Nourah bint Abdulrahman University Researchers Supporting Project number (PNURSP2025R286), Princess Nourah bint Abdulrahman University, Riyadh, Saudi Arabia.

References

1. Chaabene H, Negra Y, Bouguezzi R, Mkaouer B, Franchini E, Julio U, Hachana Y. Physical and physiological attributes of wrestlers: an update. The Journal of Strength & Conditioning Research. 2017;31(5):1411-42.
2. Kraemer WJ, Fry AC, Rubin MR, Triplett-McBride T, Gordon SE, Koziris LP, et al. Physiological and performance responses to tournament wrestling. Medicine and science in sports and exercise. 2001;33(8):1367-78.
3. Machado CLF, Nakamura FY, Fortes RP, Trapaga ID, Brusco CM, Pinto MD, Pinto RS. Changes in knee flexion-extension performance and hamstring-to-quadriceps ratio during a fatiguing isokinetic protocol in male professional futsal players. International Journal of Performance Analysis in Sport. 2023;23(4):249-63.

4. Pinto MD, Blazevich AJ, Andersen LL, Mil-Homens P, Pinto RS. Hamstring-to-quadriceps fatigue ratio offers new and different muscle function information than the conventional non-fatigued ratio. Scandinavian journal of medicine & science in sports. 2018;28(1):282-93.
5. Liu Z, Yang C, Yu J, Zhao X, Wu J, Zhang Y, et al. The effect of muscles fatigue on the knee's kinetics and kinematics characteristics. Sustainability. 2023;15(4):3029.
6. Bell J, Duke M, Travis E, Jones A. Injury Incidence and Prevalence in a Sample of Wrestlers Based in Britain: A Retrospective Study Journal of Elite Sport Performance. Journal of Elite Sport Performance. 2023;3(1):1-13.
7. Wroble R, Mysnyk M, Foster D, Albright J. Patterns of knee injuries in wrestling: a six year study. The American journal of sports medicine. 1986;14(1):55-66.
8. Croisier J-L, Ganteaume S, Binet J, Genty M, Ferret J-M. Strength imbalances and prevention of hamstring injury in professional soccer players: a prospective study. The American journal of sports medicine. 2008;36(8):1469-75.
9. Rosene JM, Fogarty TD, Mahaffey BL. Isokinetic hamstrings: quadriceps ratios in intercollegiate athletes. Journal of athletic training. 2001;36(4):378.
10. Coombs R, Garbutt G. Developments in the use of the hamstring/quadriceps ratio for the assessment of muscle balance. Journal of sports science & medicine. 2002;1(3):56.
11. Mandroukas A, Michailidis Y, Metaxas T. Muscle strength and hamstrings to quadriceps ratio in young soccer players: A cross-sectional study. Journal of Functional Morphology and Kinesiology. 2023;8(2):70.
12. Kellis E, Sahinis C, Baltzopoulos V. Is hamstrings-to-quadriceps torque ratio useful for predicting anterior cruciate ligament and hamstring injuries? A systematic and critical review. Journal of Sport and Health Science. 2023;12(3):343-58.
13. Redding J. Hamstring and Quadriceps Strength Ratio in Collegiate Wrestlers. 2022.
14. Çimen Polat S, Bulgay C, Yarım I, Cicioğlu Hİ, Çetin E. Analysis of the relationship between elite wrestlers' leg strength and balance performance, and injury history. Sports. 2018;6(2):35.
15. Han M, Akaydın Ş, Yılmaz A, Koç S. Comparison of The Physical Fitness Parameters of Special Education Vocational School Students and Inclusion High School Students. International Journal of Disabilities Sports and Health Sciences. 2024;7(3):597-607.
16. Özbay S, Ulupınar S. Strength-power tests are more effective when performed after exhaustive exercise in discrimination between top-elite and elite wrestlers. The Journal of Strength & Conditioning Research. 2022;36(2):448-54.
17. Liu C, Peng W, Qu W, Zhang Z, Sun J, He J, et al. Gender differences in the impact of fatigue on lower limb landing biomechanics and their association with anterior cruciate ligament (ACL) injuries: A systematic review and meta-analysis. PLoS One. 2025;20(5):e0321925.
18. Lord C, Ma'ayah F, Blazevich AJ. Change in knee flexor torque after fatiguing exercise identifies previous hamstring injury in football players. Scandinavian journal of medicine & science in sports. 2018;28(3):1235-43.
19. Sangnier S, Tourny-Chollet C. Comparison of the decrease in strength between hamstrings and quadriceps during isokinetic fatigue testing in semiprofessional soccer players. International journal of sports medicine. 2007;28(11):952-7.
20. Barbas I, Fatouros IG, Douroudos II, Chatzinikolaou A, Michailidis Y, Draganidis D, et al. Physiological and performance adaptations of elite Greco-Roman wrestlers during a one-day tournament. European journal of applied physiology. 2011;111(7):1421-36.
21. McKay AK, Stellingwerff T, Smith ES, Martin DT, Mujika I, Goosey-Tolfrey VL, et al. Defining training and performance caliber: a participant classification framework. International journal of sports physiology and performance. 2021;17(2):317-31.
22. Cingel v, EHR, Kleinrensink G, Rooijens P, Uitterlinden E, Aufdemkampe G, Stoeckart R. Learning effect in isokinetic testing of ankle invertors and evertors. Isokinetics and exercise science. 2001;9(4):171-7.

23. Alvares JBdAR, Rodrigues R, de Azevedo Franke R, da Silva BGC, Pinto RS, Vaz MA, Baroni BM. Inter-machine reliability of the Biodex and Cybex isokinetic dynamometers for knee flexor/extensor isometric, concentric and eccentric tests. Physical Therapy in Sport. 2015;16(1):59-65.
24. Cohen J. Statistical power analysis for the behavioral sciences (2d ed.). Hillsdale, NJ: Lawrence Earlbaum Associates. Find this resource. 1988.
25. Kolimechkov S, Makaveev R, Zaykova D, Petrov L. Tabata protocol-based high-intensity interval training in freestyle wrestlers. Pedagogy of Physical Culture and Sports. 2023;27(6):467-73.
26. Schuermans J, Van Tiggelen D, Danneels L, Witvrouw E. Susceptibility to hamstring injuries in soccer: a prospective study using muscle functional magnetic resonance imaging. The American journal of sports medicine. 2016;44(5):1276-85.
27. Moon J, Lee J, Kim K, Koo D, Lee J, Pathak P, et al. Effect of muscle-specific fatigue on the risk of anterior cruciate ligament injury in females. Applied Sciences. 2021;11(11):4969.
28. Wright J, Ball N, Wood L. Fatigue, H/Q ratios and muscle coactivation in recreational football players. Isokinetics and exercise science. 2009;17(3):161-7.
29. Baić M, Trajković N, Djordjevic D, Stankovic M, Pekas D. Strength profile in wrestlers-a systematic review. Archives of Budo. 2022;18:151.
30. Moreira PV, Gonçalves M, Crozara LF, Castro A, Neto AFA, Goethel MF, Cardozo AC. Effects of fatigue on the neuromuscular capacity of professional soccer players. Isokinetics and Exercise Science. 2015;23(4):275-82.
31. Delextrat A, Gregory J, Cohen D. The use of the functional H: Q ratio to assess fatigue in soccer. International journal of sports medicine. 2010;31(03):192-7.
32. Shirani Dastjerdi A, Esmaeili H, Sadeghi M, Bashiri B. Effect of simulated handball match-induced fatigue on isokinetic hamstring-to-quadriceps ratio and evertor-to-invertor ratio in professional players. PLoS One. 2025;20(5):e0323978.
33. Nevot-Casas O, Pujol-Marzo M, Moreno-Planes B, Fort-Vanmeerhaeghe A. Neuromuscular fatigue effects on Hamstring to Quadriceps Ratio in young female players. Apunts Sports Medicine. 2020;55(208):137-42.
34. Rahnama N, Reilly T, Lees A, Graham-Smith P. Muscle fatigue induced by exercise simulating the work rate of competitive soccer. Journal of Sports Science. 2003;21(11):933-42.
35. Hamada T, Sale D, MacDougall J, Tarnopolsky M. Interaction of fibre type, potentiation and fatigue in human knee extensor muscles. Acta physiologica scandinavica. 2003;178(2):165-73.
36. Noakes TD. Physiological models to understand exercise fatigue and the adaptations that predict or enhance athletic performance. Scandinavian Journal of Medicine & Science in Sports: Review Article. 2000;10(3):123-45.
37. Baroni BM, Ruas CV, Ribeiro-Alvares JB, Pinto RS. Hamstring-to-quadriceps torque ratios of professional male soccer players: A systematic review. The Journal of Strength & Conditioning Research. 2020;34(1):281-93.

Figure

Click here to access/download;Figure;Figure 1.png

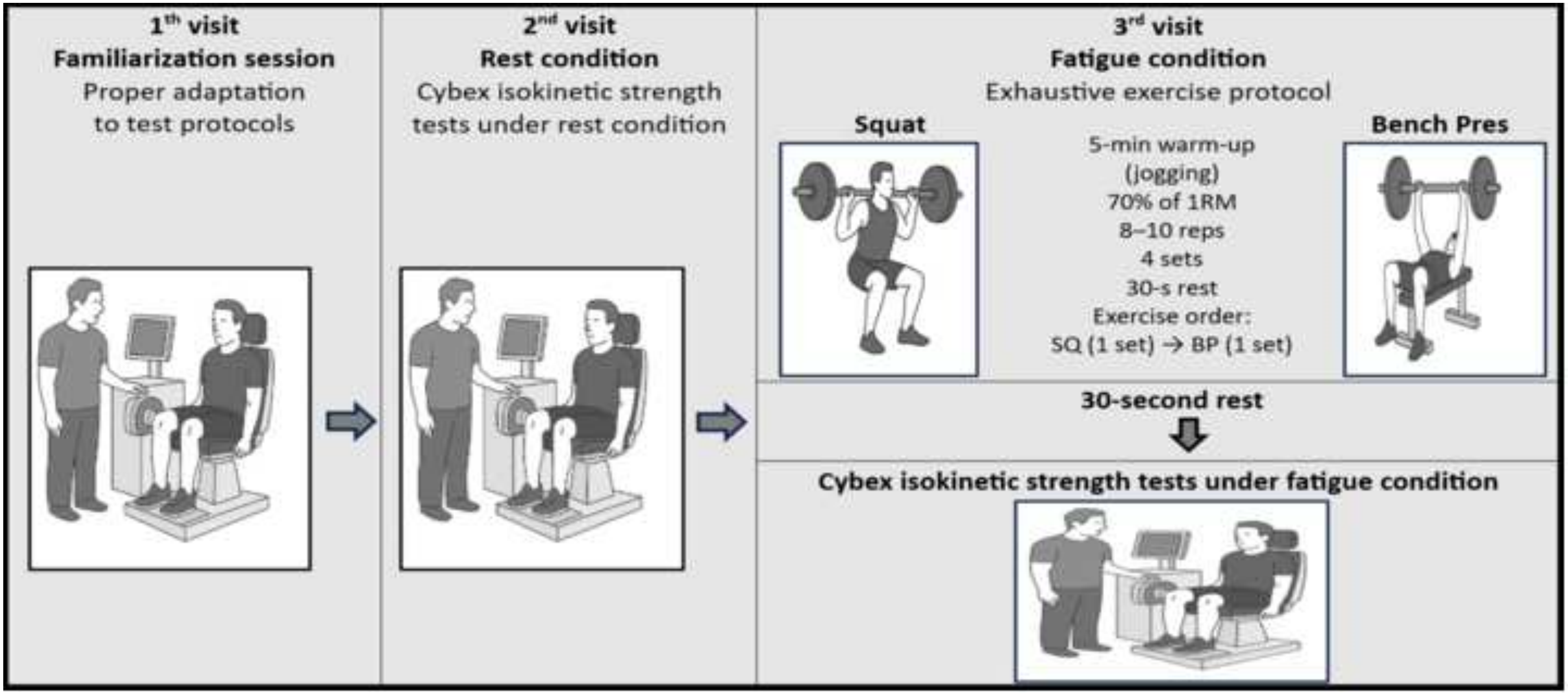

 Click here to access/download;Figure;Figure 2.tiff

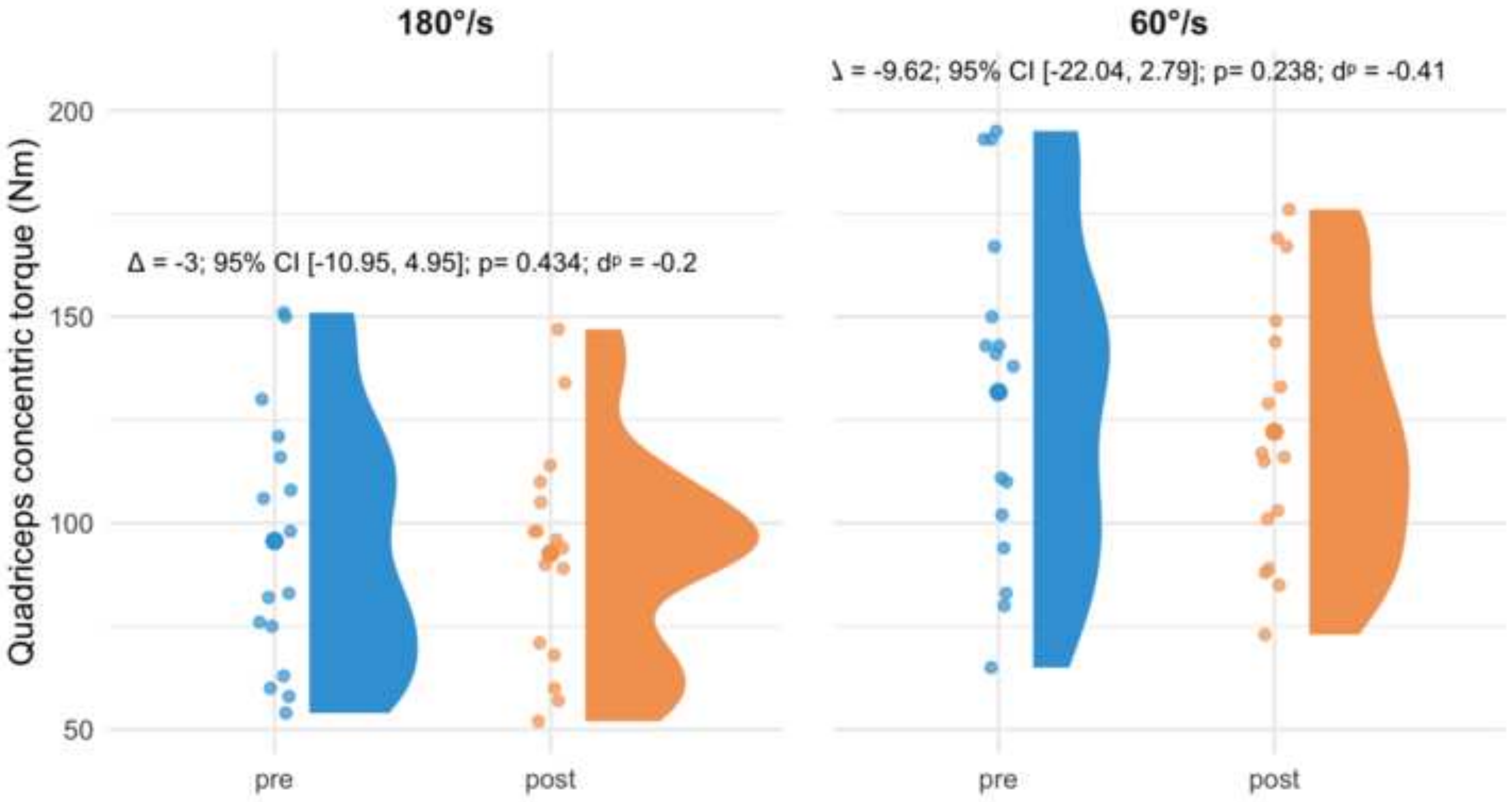

 Click here to access/download;Figure;Figure 3.tiff

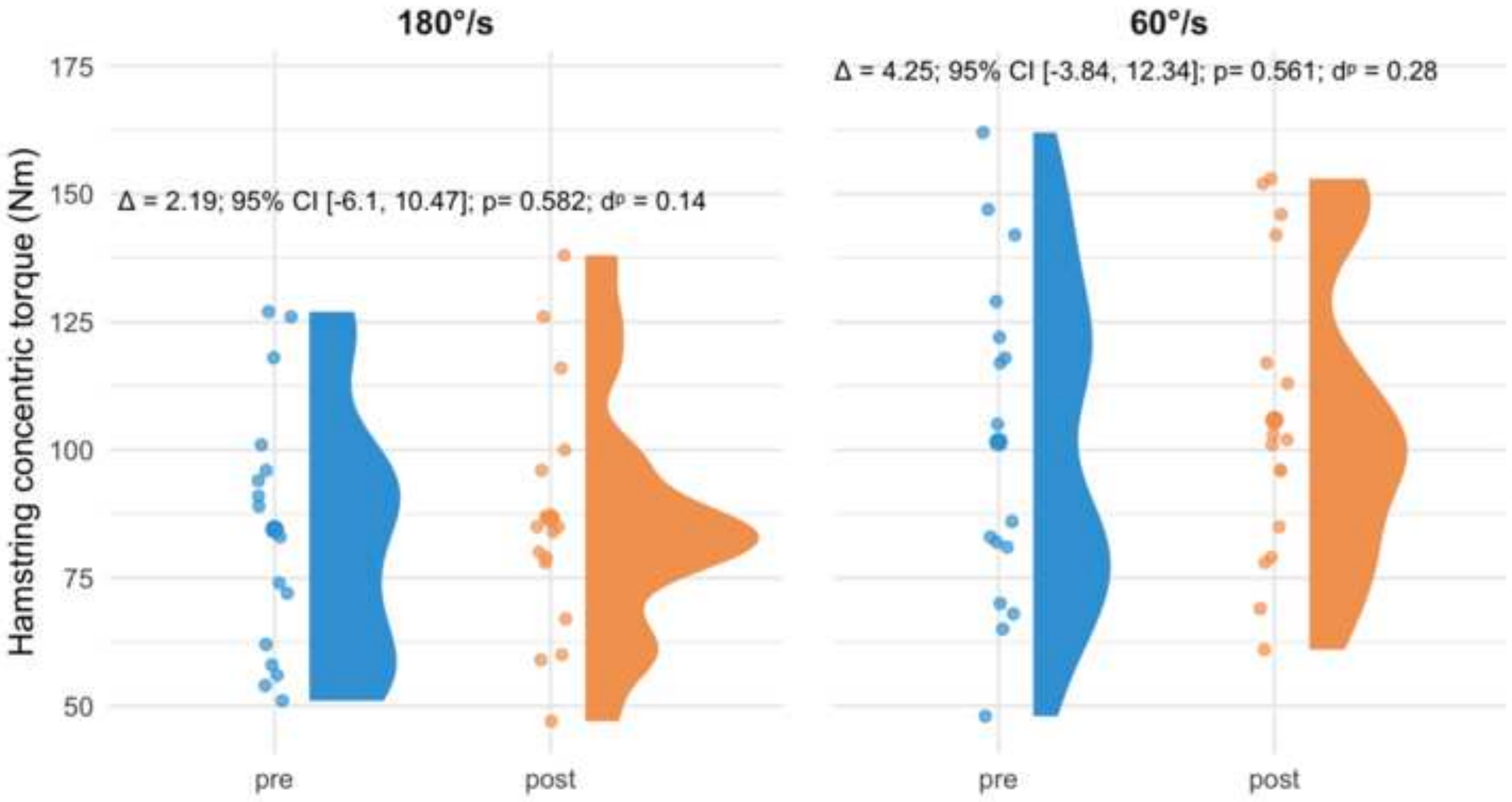

Figure Click here to access/download;Figure;Figure 4.tiff

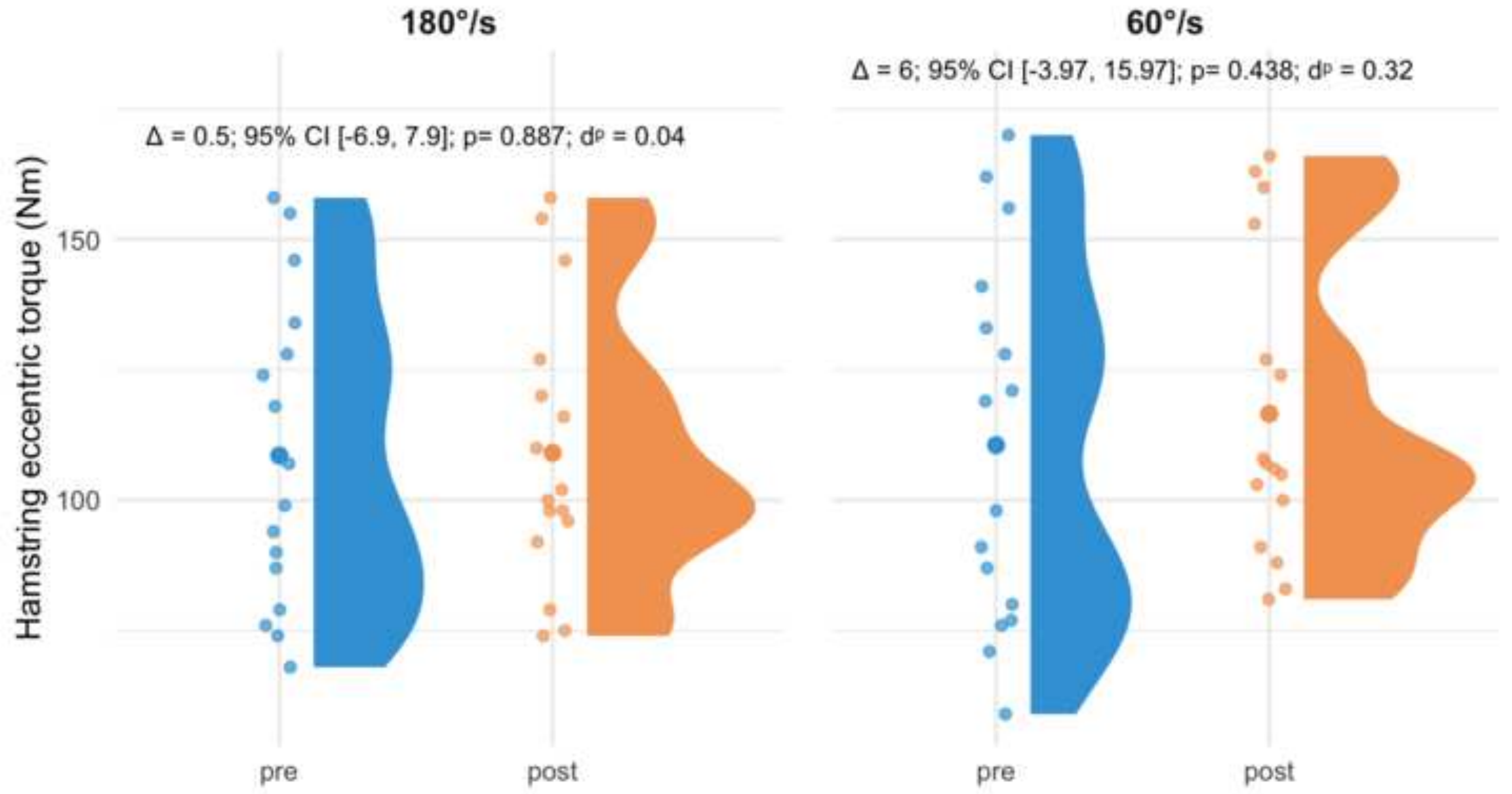

Figure

Click here to access/download;Figure;Figure 5.tiff

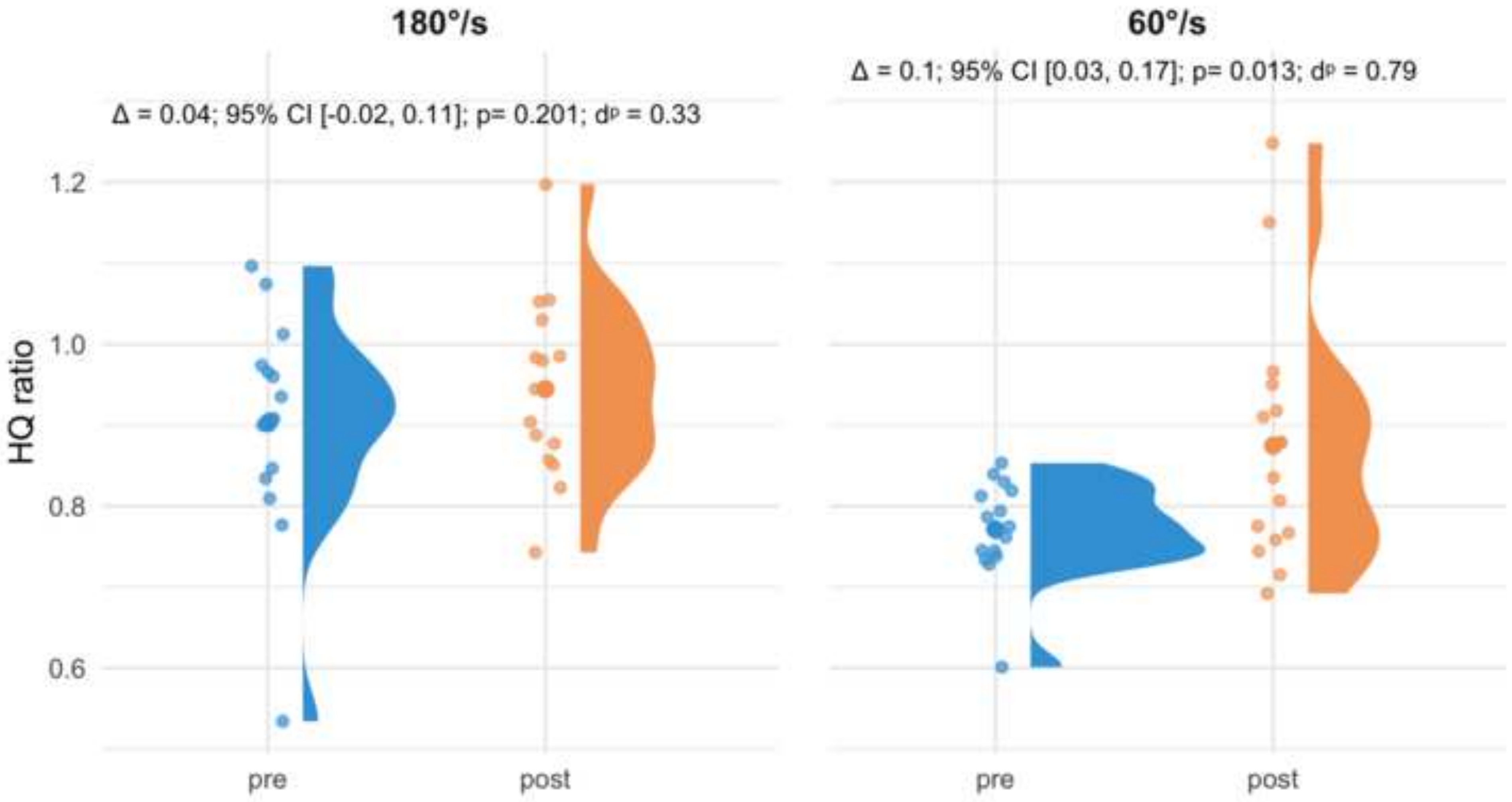

 Click here to access/download;Figure;Figure 6.tiff

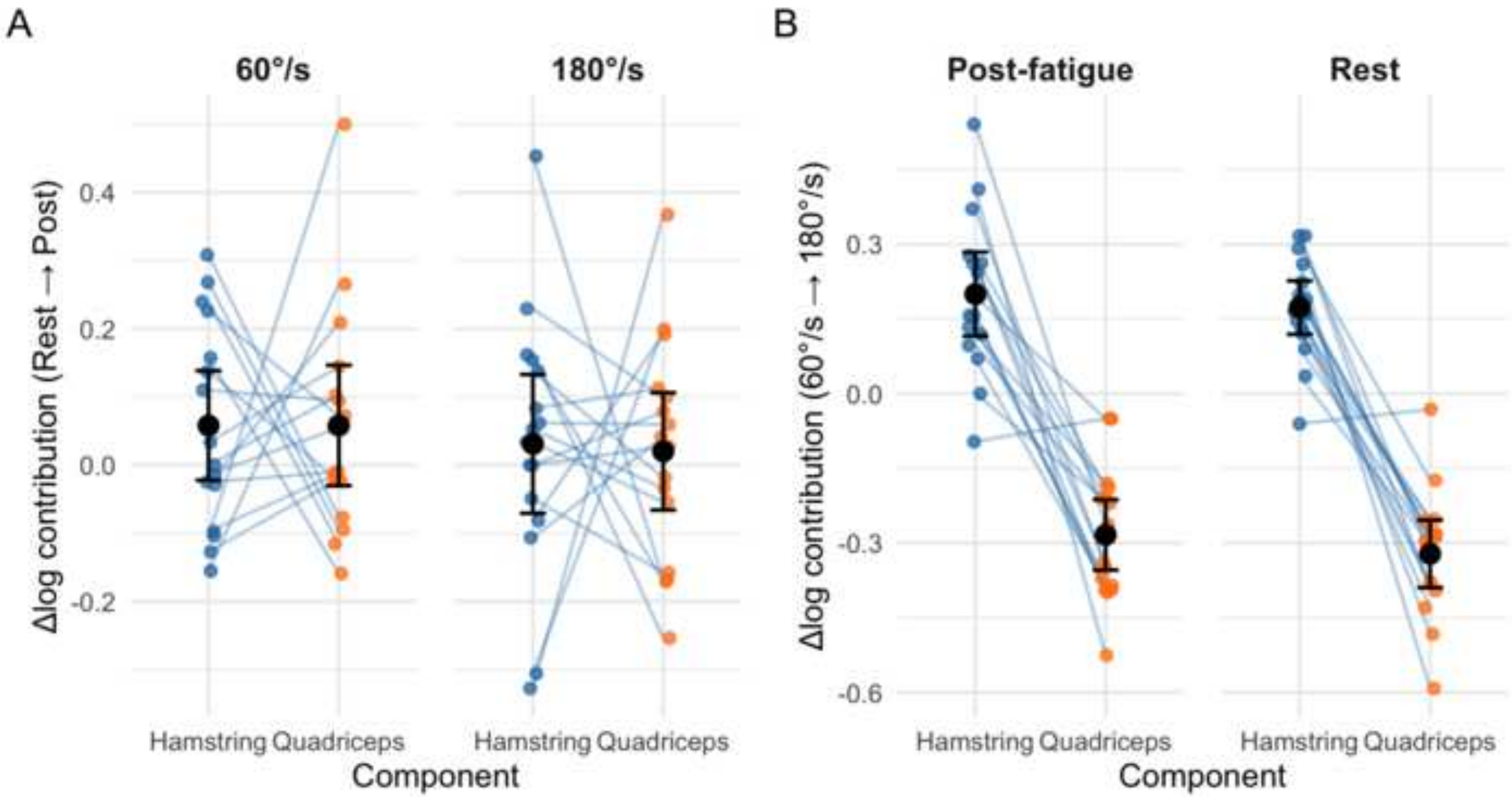

Figure
Click here to access/download;Figure;Figure 7.tiff

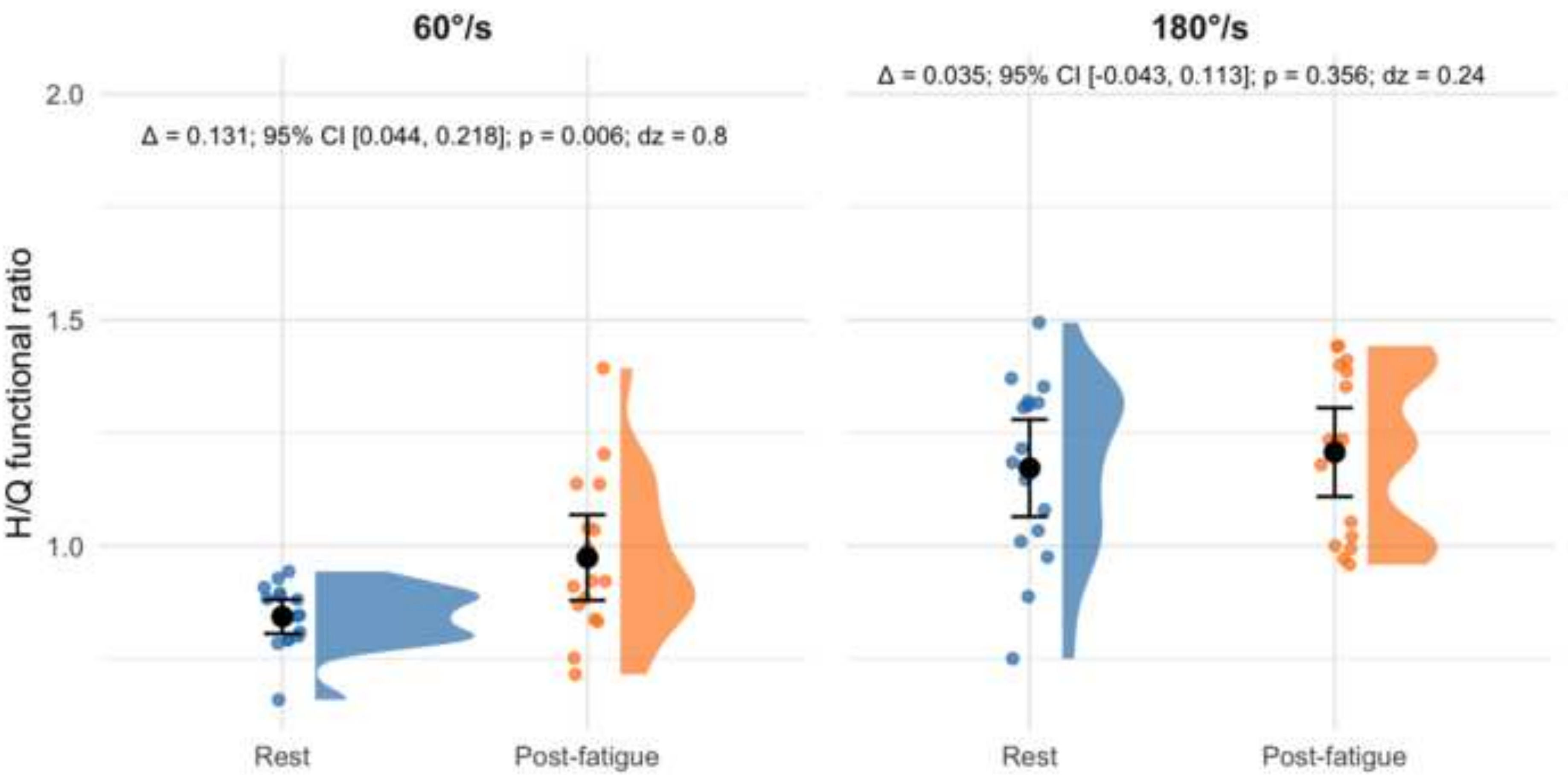

 Click here to access/download;Figure;Figure 8.tiff

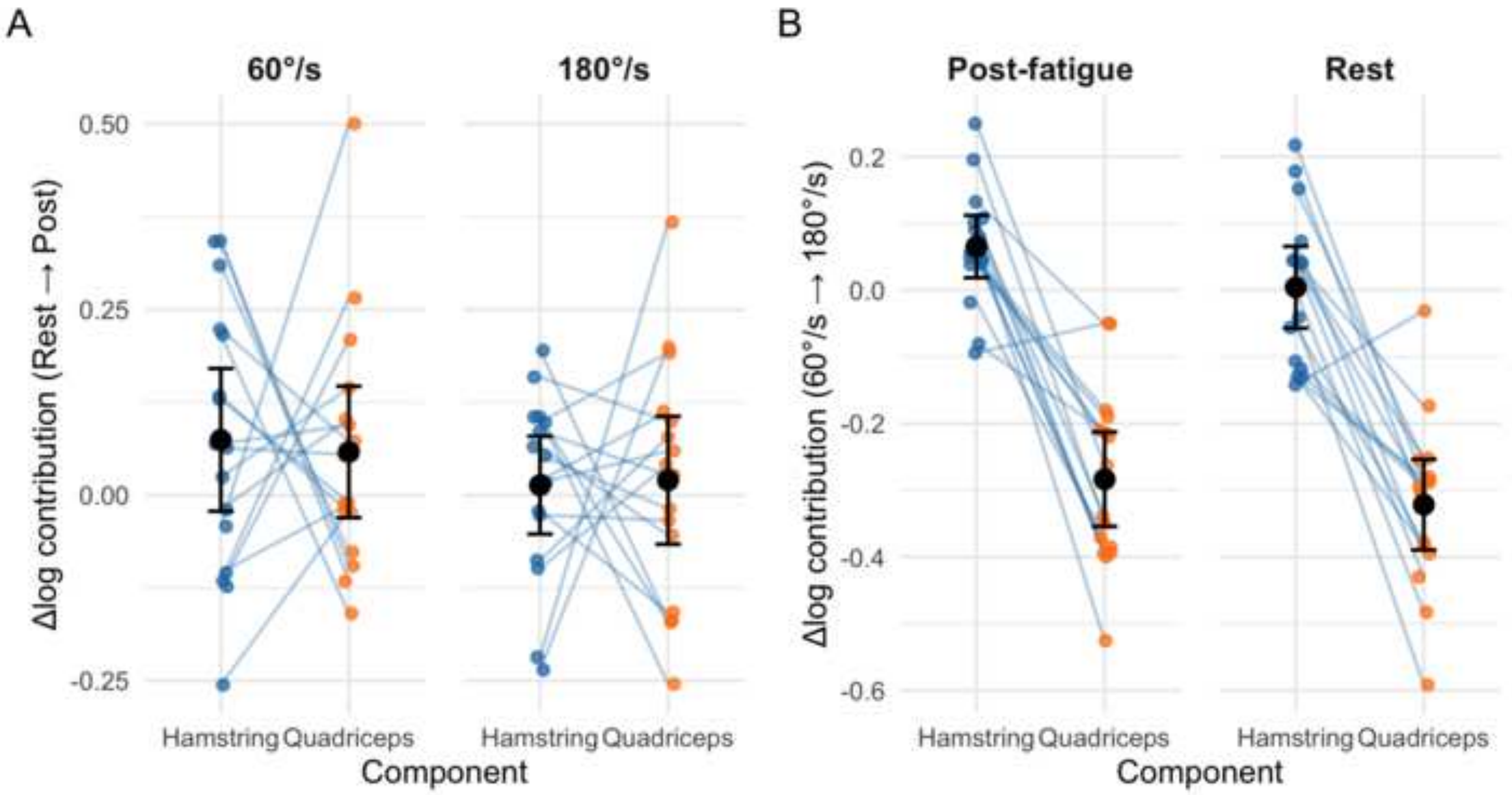

Supplement: Attachment — Submitted filename: PONE-D-25-60422_reviewer.pdf [file pone.0342975.s002.pdf]
