# Supplementary material for: Comparative analysis of isokinetic ratios between hamstrings and quadriceps in wrestlers: Resting state versus post-fatigue evaluation
Source: PLoS One. 2026 Jun 12;21(6):e0342975. doi: 10.1371/journal.pone.0342975 (PMC13262851; doi:10.1371/journal.pone.0342975)
Supplement: Attachment — Submitted filename: Reviewer Comment.docx [file pone.0342975.s003.docx]

**Manuscript number** …

**Title** Comparative Analysis of Isokinetic Ratios Between Hamstrings and Quadriceps in

Wrestlers: Resting State Versus Post-Fatigue Evaluation

Thank you for choosing me as a reviewer.

**General comment:**

Excellent work! Congratulations on a very well-written manuscript. I only have a few minor comments/suggestions.

- When using abbreviations, please first provide the full term followed by the abbreviation in parentheses (e.g., functional hamstring-to-quadriceps ratio (H:QFunc) or conventional hamstring-to-quadriceps ratio (H:QConv) and then use the abbreviation throughout the manuscript. This will improve clarity and readability for all readers.
- The introduction presents both ratios, but their practical differences and applications remain
- It is not clear which leg was tested—was it the dominant leg? If so, please clarify how dominance was defined, as the concept of a dominant leg can vary depending on the type of motor task or movement being considered. Providing this information would improve clarity and reproducibility of the protocol.
- Pre- and post-fatigue, indicating that velocities differ overall. However, this effect does not specify differences separately for pre- or post-fatigue unless the Time × Velocity interaction is significant or a simple main effects analysis is conducted. Therefore, it is recommended to perform a simple main effects or post-hoc analysis for each level of Time to clearly report how velocity changes before and after fatigue. unclear. It would be helpful if you could provide a more detailed explanation.
- It may be helpful to clarify how the peak torque was determined across the three maximal repetitions—for example, whether the single highest value or an average was used.
