# Supplementary material for: Comparative analysis of isokinetic ratios between hamstrings and quadriceps in wrestlers: Resting state versus post-fatigue evaluation
Source: PLoS One. 2026 Jun 12;21(6):e0342975. doi: 10.1371/journal.pone.0342975 (PMC13262851; doi:10.1371/journal.pone.0342975)
Supplement: Attachment — Submitted filename: R1. Respose to Reviewer 2 - Kopya.docx [file pone.0342975.s005.docx]

**REVIEWERS’ COMMENTS AND AUTHORS’ RESPONSE**

**Paper title:** Comparative Analysis of Isokinetic Ratios Between Hamstrings and Quadriceps in Wrestlers: Resting State Versus Post-Fatigue Evaluation

**Revised paper title:** Comparative Analysis of Isokinetic Ratios Between Hamstrings and Quadriceps in Wrestlers: Resting State Versus Post-Fatigue Evaluation

Dear Editor,

The authors would like to thank the reviewers for their precious time and invaluable comments. We have carefully addressed all the comments. The corresponding changes and refinements made in the revised paper are summarized in our response below. Our detailed, point-by-point responses to the reviewer comments are given below. Additionally, we have carefully revised the manuscript to ensure that the text is optimally phrased and free from typographical and grammatical errors. In the research text, corrections from the reviewer are indicated in yellow.

Thank you once again for considering our work and for inviting us to submit the revised manuscript. We look forward to hearing from you.

Best regards.

**Reviewer 2**

**Minor comments**

**1. ABSTRACT**

**Q1. -	When using abbreviations, please first provide the full term followed by the abbreviation in parentheses (e.g., functional hamstring-to-quadriceps ratio (H:QFunc) or conventional hamstring-to-quadriceps ratio (H:QConv) and then use the abbreviation throughout the manuscript. This will improve clarity and readability for all readers.**

**A1.** Thank you for your valuable comment. The manuscript has been carefully revised, and all abbreviations used in the text are now written in their full form at their first occurrence, followed by the corresponding abbreviations. Only the abbreviations are used in subsequent occurrences. This revision was made to improve the clarity and readability of the manuscript.

**2. INTRODUCTION**

**Q2. -	The introduction presents both ratios, but their practical differences and applications remain**

**A2.** The paragraph has been revised according to the reviewer’s suggestion. **“**The H/Q strength ratio can be calculated through various approaches, reflecting different dimensions of knee muscle performance [1], and it has been proposed as a potential protector against hamstring strains and anterior cruciate ligament injuries [2]. Since the H/Q ratio is an indicator of critical importance for maintaining knee joint stability, it is commonly used as one of the parameters to assess the risk for lower extremity injuries [3, 4]. The conventional H/Q ratio (H:QConv) is calculated from the concentric peak torques of the hamstring and quadriceps muscles. It reflects general agonist–antagonist strength balance and is usually assessed at low to intermediate angular velocities [1]. The H:QConv strength ratio is about 0.66 [4]. On the other hand, functional H/Q ratio (H:QFunc) is a functional measure used in injury risk assessment that describes the relationship between eccentric hamstring and concentric quadriceps peak torques [1]. The H:QFunc ratio is generally considered to be at least 1.0 or more [5]. High-speed running can be named among primary mechanisms for developing a strain into the hamstrings [6]. These injuries usually take place in the last part of the swing phase when the hamstring muscles have to produce a lot of force while lengthening [7]. Moreover, it is the co-contraction of the hamstring muscles that prevents excessive contraction of the quadriceps, thus increasing stability at the level of the knee joint and possibly attenuating excessive knee abduction moments during landing tasks [8]. Given these traits, the functional H/Q ratio is seen as offering an evaluation more akin to the mechanical conditions under which knee injuries happen [1]. It has also been proposed that this ratio might be a better measure when assessing movements specific to a sport [9]. These H:Q ratios have become common in professional teams as part of performance assessments and injury risk evaluations [10]. In wrestling, the H:Q ratio is an essential parameter for assessing performance, balance in muscle strength, and injury risk [11, 12].”

**3. METHODS**

**Q3. It is not clear which leg was tested—was it the dominant leg? If so, please clarify how dominance was defined, as the concept of a dominant leg can vary depending on the type of motor task or movement being considered. Providing this information would improve clarity and reproducibility of the protocol.**

**A3.** We thank the reviewer for the suggestion **“**All isokinetic strength assessments were performed on the dominant leg of each participant, which was defined as the preferred kicking leg.” The information was added to methods.

**Q4. -It may be helpful to clarify how the peak torque was determined across the three maximal repetitions—for example, whether** **the single highest value or an average was used.**

**A4.** We thank the reviewer for the suggestion **“**(the single highest value of 3 rep).” The information was added to methods.

**4. RESULTS**

**Q5. Pre- and post-fatigue, indicating that velocities differ overall. However, this effect does not specify differences separately for pre- or post-fatigue unless the Time × Velocity interaction is significant or a simple main effects analysis is conducted. Therefore, it is recommended to perform a simple main effects or post-hoc analysis for each level of Time to clearly report how velocity changes before and after fatigue. unclear. It would be helpful if you could provide a more detailed explanation.**

**A5.** We appreciate your valuable feedback and clear guidance. Following your suggestion, we conducted simple main effects analyses in addition to the ANOVA for the two-factor repeated measures and reported the results with Holm correction, 95% CI, and effect size information. The analyses were conducted as the effect of Velocity on each Time level (Velocity | Time) and the effect of Time on each Velocity level (Time | Velocity). The methods section has been updated, and a summary of the results has been added to the footnote of Table 2.

“To clarify the interpretation of the interaction, simple main effects were tested using emmeans following RM-ANOVA (Speed | Time and Time | Speed), comparisons were corrected using Holm's method, and results were reported using Cohen's paired d (dᶻ) with 95% CI.”

“We additionally tested simple main effects (Holm-adjusted) of Velocity within each level of Time and of Time within each Velocity. For QCON, the within-speed time contrasts reproduced the same direction but did not reach significance at either speed (60°/s: Rest−Post = +9.62 Nm, 95% CI [−2.79, 22.00], p = .119; 180°/s: +3.00 Nm, [−4.95, 11.00], p = .434). For HCON, Velocity was significantly higher at 60°/s than 180°/s both at rest and post-fatigue (Rest: +17.1 Nm, [+10.8, +23.3], p < .001; Post: +19.1 Nm, [+10.4, +27.8], p < .001), whereas the Time simple effects were not significant within either speed (60°/s: Rest−Post = −4.25 Nm, [−12.3, 3.84], p = .281; 180°/s: −2.19 Nm, [−10.5, 6.10], p= .582). For HECC, the Velocity simple effect was significant only post-fatigue (Post: +7.5 Nm, [+1.81, +13.19], p = .013), with no significant time contrasts within speeds. For H:QConv, the Time simple effect was significant at 60°/s(Rest−Post = −0.104, [−0.174, −0.034], p = .006), but not at 180°/s (−0.042, [−0.109, 0.025], p = .201); the Velocity simple effect was significant at rest (−0.132, [−0.185, −0.078], p < .001). For H:QFunc, the Time×Velocity interaction was clarified by a significant time contrast at 60°/s (Rest−Post = −0.131, [−0.217, −0.044], p = .006) but not at 180°/s(−0.035, [−0.114, 0.043], p = .356), while the Velocity simple effects were significant at both time levels (Rest: −0.329, [−0.430, −0.228], p < .001; Post: −0.233, [−0.312, −0.154], p < .001). These simple-effects results make explicit how velocity behaves within rest and post-fatigue and confirm the omnibus ANOVA conclusions.”
