# Supplementary material for: Comparative analysis of isokinetic ratios between hamstrings and quadriceps in wrestlers: Resting state versus post-fatigue evaluation
Source: PLoS One. 2026 Jun 12;21(6):e0342975. doi: 10.1371/journal.pone.0342975 (PMC13262851; doi:10.1371/journal.pone.0342975)
Supplement: Peer review history

# Peer review: Decision Letter 0

**DOI:** 10.1371/journal.pone.0342975.r001

**Authors:** Esedullah Akaras

17 Dec 2025

PONE-D-25-60422

Comparative Analysis of Isokinetic Ratios Between Hamstrings and Quadriceps in Wrestlers: Resting State Versus Post-Fatigue Evaluation

PLOS One

Dear Dr. Kurtoğlu,

Thank you for submitting your manuscript to PLOS ONE. After careful consideration, we feel that it has merit but does not fully meet PLOS ONE’s publication criteria as it currently stands. Therefore, we invite you to submit a revised version of the manuscript that addresses the points raised during the review process.

Please submit your revised manuscript by Jan 31 2026 11:59PM. If you will need more time than this to complete your revisions, please reply to this message or contact the journal office at plosone@plos.org. When you're ready to submit your revision, log on to https://www.editorialmanager.com/pone/ and select the 'Submissions Needing Revision' folder to locate your manuscript file.

Please include the following items when submitting your revised manuscript:

- A letter that responds to each point raised by the academic editor and reviewer(s). You should upload this letter as a separate file labeled 'Response to Reviewers'.
- A marked-up copy of your manuscript that highlights changes made to the original version. You should upload this as a separate file labeled 'Revised Manuscript with Track Changes'.
- An unmarked version of your revised paper without tracked changes. You should upload this as a separate file labeled 'Manuscript'.

If you would like to make changes to your financial disclosure, please include your updated statement in your cover letter. Guidelines for resubmitting your figure files are available below the reviewer comments at the end of this letter.

If applicable, we recommend that you deposit your laboratory protocols in protocols.io to enhance the reproducibility of your results. Protocols.io assigns your protocol its own identifier (DOI) so that it can be cited independently in the future. For instructions see: https://journals.plos.org/plosone/s/submission-guidelines#loc-laboratory-protocols. Additionally, PLOS ONE offers an option for publishing peer-reviewed Lab Protocol articles, which describe protocols hosted on protocols.io. Read more information on sharing protocols at https://plos.org/protocols?utm_medium=editorial-email&utm_source=authorletters&utm_campaign=protocols.

We look forward to receiving your revised manuscript.

Kind regards,

Esedullah Akaras

Academic Editor

PLOS One

Journal requirements:

When submitting your revision, we need you to address these additional requirements.

1. Please ensure that your manuscript meets PLOS ONE's style requirements, including those for file naming. The PLOS ONE style templates can be found at

https://journals.plos.org/plosone/s/file?id=wjVg/PLOSOne_formatting_sample_main_body.pdf and

https://journals.plos.org/plosone/s/file?id=ba62/PLOSOne_formatting_sample_title_authors_affiliations.pdf

2. To comply with PLOS One submissions requirements, in your Methods section, please provide additional information regarding the experiments involving animals and ensure you have included details on (1) methods of sacrifice, (2) methods of anesthesia and/or analgesia, and (3) efforts to alleviate suffering.

3. Thank you for stating the following in the Acknowledgments Section of your manuscript:

“We extend our gratitude to the participants for their invaluable contribution to this study. Special thanks to the Princess Nourah bint Abdulrahman University Researchers Supporting Project number (PNURSP2025R286), Princess Nourah bint Abdulrahman University, Riyadh, Saudi Arabia.”

We note that you have provided funding information that is not currently declared in your Funding Statement. However, funding information should not appear in the Acknowledgments section or other areas of your manuscript. We will only publish funding information present in the Funding Statement section of the online submission form.

Please remove any funding-related text from the manuscript and let us know how you would like to update your Funding Statement. Currently, your Funding Statement reads as follows:

“The author(s) received no specific funding for this work.”

Please include your amended statements within your cover letter; we will change the online submission form on your behalf.

4. In the online submission form you indicate that your data is not available for proprietary reasons and have provided a contact point for accessing this data. Please note that your current contact point is a co-author on this manuscript. According to our Data Policy, the contact point must not be an author on the manuscript and must be an institutional contact, ideally not an individual. Please revise your data statement to a non-author institutional point of contact, such as a data access or ethics committee, and send this to us via return email. Please also include contact information for the third party organization, and please include the full citation of where the data can be found.

5. Your ethics statement should only appear in the Methods section of your manuscript. If your ethics statement is written in any section besides the Methods, please delete it from any other section.

6. Please remove your figures from within your manuscript file, leaving only the individual TIFF/EPS image files, uploaded separately. These will be automatically included in the reviewers’ PDF.

If the reviewer comments include a recommendation to cite specific previously published works, please review and evaluate these publications to determine whether they are relevant and should be cited. There is no requirement to cite these works unless the editor has indicated otherwise.

[Note: HTML markup is below. Please do not edit.]

Reviewers' comments:

Reviewer's Responses to Questions

**Comments to the Author**

1. Is the manuscript technically sound, and do the data support the conclusions?

The manuscript must describe a technically sound piece of scientific research with data that supports the conclusions. Experiments must have been conducted rigorously, with appropriate controls, replication, and sample sizes. The conclusions must be drawn appropriately based on the data presented.

Reviewer #1: Yes

Reviewer #2: Yes

**********

2. Has the statistical analysis been performed appropriately and rigorously?

Reviewer #1: Yes

Reviewer #2: Yes

**********

3. Have the authors made all data underlying the findings in their manuscript fully available?

The PLOS Data policy requires authors to make all data underlying the findings described in their manuscript fully available without restriction, with rare exception (please refer to the Data Availability Statement in the manuscript PDF file). The data should be provided as part of the manuscript or its supporting information, or deposited to a public repository. For example, in addition to summary statistics, the data points behind means, medians and variance measures should be available. If there are restrictions on publicly sharing data—e.g. participant privacy or use of data from a third party—those must be specified.

Reviewer #1: Yes

Reviewer #2: Yes

**********

4. Is the manuscript presented in an intelligible fashion and written in standard English?

PLOS ONE does not copyedit accepted manuscripts, so the language in submitted articles must be clear, correct, and unambiguous. Any typographical or grammatical errors should be corrected at revision, so please note any specific errors here.

Reviewer #1: Yes

Reviewer #2: Yes

**********

5. Review Comments to the Author

Please use the space provided to explain your answers to the questions above. You may also include additional comments for the author, including concerns about dual publication, research ethics, or publication ethics. (Please upload your review as an attachment if it exceeds 20,000 characters)

Reviewer #1: Dear Authors. Thank you for your work. I am surprised after reading your work. I live in a country which is a Trendsetting Country in wrestling. I carefully evaluated your work and have some concerns which are commented to the file.

Reviewer #2: Manuscript number …

Title Comparative Analysis of Isokinetic Ratios Between Hamstrings and Quadriceps in

Wrestlers: Resting State Versus Post-Fatigue Evaluation

Thank you for choosing me as a reviewer.

General comment:

Excellent work! Congratulations on a very well-written manuscript. I only have a few minor comments/suggestions.

- When using abbreviations, please first provide the full term followed by the abbreviation in parentheses (e.g., functional hamstring-to-quadriceps ratio (H:QFunc) or conventional hamstring-to-quadriceps ratio (H:QConv) and then use the abbreviation throughout the manuscript. This will improve clarity and readability for all readers.

- The introduction presents both ratios, but their practical differences and applications remain

- It is not clear which leg was tested—was it the dominant leg? If so, please clarify how dominance was defined, as the concept of a dominant leg can vary depending on the type of motor task or movement being considered. Providing this information would improve clarity and reproducibility of the protocol.

- Pre- and post-fatigue, indicating that velocities differ overall. However, this effect does not specify differences separately for pre- or post-fatigue unless the Time × Velocity interaction is significant or a simple main effects analysis is conducted. Therefore, it is recommended to perform a simple main effects or post-hoc analysis for each level of Time to clearly report how velocity changes before and after fatigue. unclear. It would be helpful if you could provide a more detailed explanation.

- It may be helpful to clarify how the peak torque was determined across the three maximal repetitions—for example, whether the single highest value or an average was used.

**********

6. PLOS authors have the option to publish the peer review history of their article (what does this mean?). If published, this will include your full peer review and any attached files.

If you choose “no”, your identity will remain anonymous but your review may still be made public.

**Do you want your identity to be public for this peer review?**  For information about this choice, including consent withdrawal, please see our Privacy Policy.

Reviewer #1: No

Reviewer #2: **Yes:**  Mohamadreza Hatefi

**********

[NOTE: If reviewer comments were submitted as an attachment file, they will be attached to this email and accessible via the submission site. Please log into your account, locate the manuscript record, and check for the action link "View Attachments". If this link does not appear, there are no attachment files.]

To ensure your figures meet our technical requirements, please review our figure guidelines: https://journals.plos.org/plosone/s/figures

You may also use PLOS’s free figure tool, NAAS, to help you prepare publication quality figures: https://journals.plos.org/plosone/s/figures#loc-tools-for-figure-preparation

NAAS will assess whether your figures meet our technical requirements by comparing each figure against our figure specifications.

Attachment Submitted filename: PONE-D-25-60422_reviewer.pdf

Attachment Submitted filename: Reviewer Comment.docx

# Author comment: Author response to Decision Letter 1

**DOI:** 10.1371/journal.pone.0342975.r002

7 Jan 2026

REVIEWERS’ COMMENTS AND AUTHORS’ RESPONSE

Paper title: Comparative Analysis of Isokinetic Ratios Between Hamstrings and Quadriceps in Wrestlers: Resting State Versus Post-Fatigue Evaluation

Revised paper title: Comparative Analysis of Isokinetic Ratios Between Hamstrings and Quadriceps in Wrestlers: Resting State Versus Post-Fatigue Evaluation

Dear Editor,

The authors would like to thank the reviewers for their precious time and invaluable comments. We have carefully addressed all the comments. The corresponding changes and refinements made in the revised paper are summarized in our response below. Our detailed, point-by-point responses to the reviewer comments are given below. Additionally, we have carefully revised the manuscript to ensure that the text is optimally phrased and free from typographical and grammatical errors. In the research text, corrections from the reviewer are indicated in yellow.

Thank you once again for considering our work and for inviting us to submit the revised manuscript. We look forward to hearing from you.

Best regards.

Reviewer 1

Major comments

1. ABSTRACT

Q1. Start with a background, then state the aim.

A1. In line with your suggestion, background added to abstract. “Wrestling has high-intensity competitive demands, and tournament structures typically require athletes to compete in multiple matches within a single day. This results in accumulated neuromuscular fatigue that may disrupt the balance between hamstring and quadriceps strength, potentially increasing the risk of knee injuries.”

Q2. The participants are very young and I do think that your results cant be interpreted for general population. Their style (Greco-Roman) must be discussed in the introductory section so that why you have considered them.

A2. Approximately 60% of knee injuries have been reported to occur through non-contact mechanisms, which are commonly associated with movements that impose high loads on the knee joint, such as sudden deceleration, rapid changes of direction, and jumping–landing tasks (Belozo et al., 2024). In this context, the hamstring–quadriceps (H/Q) strength ratio is considered a critical neuromuscular factor influencing knee stability. In freestyle wrestling, frequent direct contact with the lower extremities makes it difficult to clearly distinguish whether knee injuries occur through contact or non-contact mechanisms. In contrast, Greco-Roman wrestling prohibits attacks below the waist, thereby limiting direct lower-limb contact and increasing the relative contribution of non-contact mechanisms to knee injuries. Therefore, Greco-Roman wrestlers were selected in the present study to more clearly examine fatigue-induced changes in hamstring–quadriceps strength balance. In line with your suggestion, information added to intro. “Knee injuries, especially anterior cruciate ligament injuries, are said to happen about 60% of the time in non-contact ways. These injuries are often linked with quick stops, fast turns, and jumping-landing activities that put great stress on the knee joint [8]. In this situation, the hamstring-quadriceps strength ratio is seen as an important neuromuscular factor that affects stability in the knee. In Greco-Roman wrestling, attacks below the waist are prohibited, which limits direct lower-extremity contact [9]. In contrast, freestyle wrestling allows contact with the legs and lower extremities [9], making contact-related mechanisms more prevalent. Therefore, non-contact injury mechanisms are considered more likely to contribute to knee loading patterns observed in Greco-Roman wrestling”.

2. INTRODUCTION

Q3. Providing evidence of knee injuries in end-stage competition conditions or final matches can lead to a better rationale and make better sense for the issue.

A3. We thank the reviewer for this valuable comment. We agree that providing evidence of knee injuries occurring during end-stage competition conditions or final matches could strengthen the rationale of the study. However, to the best of our knowledge, the existing literature does not report knee injury occurrence according to match order or tournament stage in wrestling competitions. Based on the reviewer’s suggestion, we have added a statement to the manuscript acknowledging this limitation of the current literature and clarifying the context of our study. “To the best of our knowledge, the existing literature does not report injury occurrence according to match order or tournament stage in wrestling competitions”.

3. METHODS

Q4. Your participants are too young. You can interpret your results only to young Greco-Roman wrestlers.

Their weight grade is controlled?

A4. We thank the reviewer for this valuable comment. We agree that, due to the relatively young age of the participants, the findings of the present study should be interpreted specifically within the context of young Greco-Roman wrestlers. Based on the reviewer’s comment, we have added this point to the Limitations section, clarifying that the results should not be generalized to adult or general wrestling populations. “This study examined the effects of a fatigue-inducing protocol on the muscle strength profiles of youth Greco-Roman wrestlers”.” Thus, both fatigue and movement velocity should be considered when developing training strategies and injury prevention programs for youth Greco-Roman wrestlers”. “First, because of the relatively small sample size, these findings cannot be generalized to the greater wrestling community or athletes from other sports.”

The sentence was added to methods “Weight category was not controlled as a separate analytical variable”. Although the athletes competed in different official weight categories, weight category was not controlled as a separate analytical variable because the analyses were based on within-subject comparisons between rest and post-fatigue conditions. The athletes included in this study were enrolled in state-operated national training camps organized by the Ministry of Youth and Sports and were part of structured Olympic preparation programs. Participants competed in officially regulated weight categories and were evaluated within their respective weight classes. In addition, all athletes trained and lived under standardized conditions within the same camp environment, including supervised training loads, nutrition, and recovery routines. These controlled conditions minimize potential weight- and lifestyle-related confounding factors and strengthen the internal validity of the study.”

Q5. Your participants are relatively novice. It is another limitation which must be acknowledged in the limitations section

A5. We thank the reviewer for this important comment. We agree that the relatively novice status of the participants represents an additional limitation of the present study. In line with the reviewer’s suggestion, this point has been acknowledged and explicitly added to the Limitations section of the manuscript. “Another limitation of this study is that the participants were relatively early in their competitive development, which may limit the generalizability of the findings to more experienced or elite adult Greco-Roman wrestlers”

Q6. Add technical aspects of the device.

A6. We thank the reviewer for the suggestion. The model and manufacturer details of the Cybex isokinetic dynamometer (Cybex NORM®, HUMAC, CA, USA) have already been specified in the Materials and Methods section.

Q7. What do you mean standardized laboratory conditions? Please clarify

A7. All measurements were conducted in the same laboratory under controlled environmental conditions, at similar times of day. The isokinetic dynamometer setup, participant positioning, warm-up protocol, and testing procedures were standardized and applied consistently for all participants by the same experienced examiner.

Q8. Which leg was assessed?

A8. We thank the reviewer for this comment. In accordance with the suggestion, the manuscript has been revised to clarify that all isokinetic strength assessments were performed on the dominant leg, defined as the preferred kicking leg.

Q9. Provide the rationale for chosing these two velocities.

A9. The angular velocities of 60°/s and 180°/s were selected as they are among the most commonly used testing speeds in isokinetic H/Q assessments and allow evaluation of muscle function under both low and moderate-velocity conditions, as reported in previous literatüre. Also, the angular velocities of 60°/s and 180°/s were selected as they are the most commonly reported speeds in the literature for assessing H/Q ratios, including systematic reviews and meta-analyses in combat sports (Rašković, S., et al. 2023). The information was added to text. “These angular velocities were selected because they are widely used in the literature to assess H/Q strength balance at low and moderate movement speeds [29].”

Q10. You can use the following reference: Esmaeili, H. and Sharifi, A., 2022. Bilateral asymmetry of hamstring to quadriceps isokinetic torque ratio in different angular velocities in professional basketball players. The Knee, 37, pp.3-9.

A10. It was added.

Q11. As like as other references

A11. It was fixed.

Q12. Holm-Bonferroni ???????

A12. As this criticism was also made by the previous referee, it was added to the text and highlighted in yellow.

“We additionally tested simple main effects (Holm-adjusted) of Velocity within each level of Time and of Time within each Velocity. For QCON, the within-speed time contrasts reproduced the same direction but did not reach significance at either speed (60°/s: Rest−Post = +9.62 Nm, 95% CI [−2.79, 22.00], p = .119; 180°/s: +3.00 Nm, [−4.95, 11.00], p = .434). For HCON, Velocity was significantly higher at 60°/s than 180°/s both at rest and post-fatigue (Rest: +17.1 Nm, [+10.8, +23.3], p < .001; Post: +19.1 Nm, [+10.4, +27.8], p < .001), whereas the Time simple effects were not significant within either speed (60°/s: Rest−Post = −4.25 Nm, [−12.3, 3.84], p = .281; 180°/s: −2.19 Nm, [−10.5, 6.10], p= .582). For HECC, the Velocity simple effect was significant only post-fatigue (Post: +7.5 Nm, [+1.81, +13.19], p = .013), with no significant time contrasts within speeds. For H:QConv, the Time simple effect was significant at 60°/s(Rest−Post = −0.104, [−0.174, −0.034], p = .006), but not at 180°/s (−0.042, [−0.109, 0.025], p = .201); the Velocity simple effect was significant at rest (−0.132, [−0.185, −0.078], p < .001). For H:QFunc, the Time×Velocity interaction was clarified by a significant time contrast at 60°/s (Rest−Post = −0.131, [−0.217, −0.044], p = .006) but not at 180°/s(−0.035, [−0.114, 0.043], p = .356), while the Velocity simple effects were significant at both time levels (Rest: −0.329, [−0.430, −0.228], p < .001; Post: −0.233, [−0.312, −0.154], p < .001). These simple-effects results make explicit how velocity behaves within rest and post-fatigue and confirm the omnibus ANOVA conclusions.”

Q13. Δlog(HKON)

A13. It was fixed.

4. RESULTS

Q14. Present as normalized to Body mass to make comparison with other studies?????????

A14. Dear Reviewer, we thank you for your suggestion regarding comparability based on body mass. While maintaining the main analysis plan, we additionally (i) analysed torques in Nm·kg⁻¹ and allometric (T/mass^b, where the coefficient b was estimated using log–log regression in the sample) form; (ii) constructed repeated measures ANCOVA models including weight as a covariate and reported simple effects adjusted for weight using emmeans. These sensitivity analyses showed that the direction and magnitude of the main findings did not change. Therefore, we added the corresponding method to the statistical analysis section.

“For sensitivity purposes, torques were additionally normalised according to body mass (Nm·kg⁻¹) and analysed allometrically as T/mass^b (b estimated via log–log regression in the sample); repeated measures ANCOVA (simple effects adjusted for mass using emmeans) were also conducted, including mass as a covariate.”

5. DISCUSSION

Q15. Kraemer et al is correct. Please note that you have only considered youth Greco-Roman wrestlers. You must take into account through the discussion.

A15. “In this study, conducted in youth Greco-Roman wrestlers, a similar phenomenon seems to explain the increase in the H:QConv and H:QFunc ratios after fatigue events”. “After fatigue, both the H:QConv and H:QFunc ratios increased in youth Greco-Roman wrestlers, mainly due to a significant decrease in quadriceps strength, while the hamstrings remained relatively unchanged”. “In contrast to the findings observed in youth Greco-Roman wrestlers in the present study, fatigue has been reported to affect the hamstrings more severely in sports such as soccer, futsal, basketball, and handball, thereby decreasing the H:Q ratio”. “This study examined the effects of a fatigue-inducing protocol on the muscle strength profiles of youth Greco-Roman wrestlers.” Were added to discussion.

Q16. This is on the soccer players. Please use it correctly.

A16. The sentence correctly rewritten again in form of your suggestion. “Fatigue has been associated with impaired muscle function and an increased risk of hamstring strain, particularly in soccer players [35], as well as anterior cruciate ligament injuries [36]”.

Q17. Use correct style for refering. Look jPONE guidelines

A17. Thank you for the comment. The manuscript has been revised, and all citations have been carefully checked and adjusted in accordance with the journal’s citation guidelines. In particular, references referring to studies conducted in other sports have been clarified and used in their appropriate context.

Q18. Discuss about effect sizes.

A18. This paragraph was rewritten in accordance of your suggestion. “In this study, both the H:QConv and H:QFunc ratios increased after fatigue. However, only the functional ratio showed a significant time×velocity interaction, indicating greater sensitivity to changes in both fatigue and contraction angular velocity. The effect size analysis revealed a large effect of fatigue on the H:QFunc ratio, whereas the effects observed for the H:QConv ratio were comparatively smaller and less sensitive to velocity-related changes. This is consistent with previous studies that identified the H:QFunc ratio as a more robust indicator of neuromuscular balance and injury risk [4]. Croisier et al reported that reduced H:QFunc ratios represent one of the most common neuromuscular imbalances in athletes and are better predictors of injury risk than reduced H:QConv ratios [10]. Baroni et al. also emphasized that the H:QFunc ratio more accurately reflects sport-specific muscle coordination demands, particularly at higher angular velocities [42]. Overall, the presence of larger and velocity-dependent effects for the H:QFunc ratio supports its greater practical relevance compared with the conventional ratio. These results highlight the importance of assessing the H:QFunc ratio, especially under conditions of fatigue and varying angular velocities, as a more informative and practical approach for monitoring muscular balance and injury susceptibility in athletes.”

6. CONCLUSION

Q19. You can only generalize your results for young Greco-Roman wrestlers. Please acknowledge it.

A19. Thank you for this comment. We have revised the Conclusions section to explicitly acknowledge that the findings of the present study are specific to youth Greco-Roman wrestlers and should not be generalized to adult or elite wrestling populations. “However, these findings should be interpreted with caution, as they are specific to youth Greco-Roman wrestlers and may not be directly generalizable to adult or elite wrestling populations”.

Reviewer 2

Minor comments

1. ABSTRACT

Q1. - When using abbreviations, please first provide the full term followed by the abbreviation in parentheses (e.g., functional hamstring-to-quadriceps ratio (H:QFunc) or conventional hamstring-to-quadriceps ratio (H:QConv) and then use the abbreviation throughout the manuscript. This will improve clarity and readability for all readers.

A1. Thank you for your valuable comment. The manuscript has been carefully revised, and all abbreviations used in the text are now written in their full form at

Attachment Submitted filename: R1. Respose to Reviewer 2 - Kopya.docx

# Peer review: Decision Letter 1

**DOI:** 10.1371/journal.pone.0342975.r003

**Authors:** Esedullah Akaras

2 Feb 2026

<p>Comparative Analysis of Isokinetic Ratios Between Hamstrings and Quadriceps in Wrestlers: Resting State Versus Post-Fatigue Evaluation

PONE-D-25-60422R1

Dear Dr. Kurtoğlu,

We’re pleased to inform you that your manuscript has been judged scientifically suitable for publication and will be formally accepted for publication once it meets all outstanding technical requirements.

Within one week, you’ll receive an e-mail detailing the required amendments. When these have been addressed, you’ll receive a formal acceptance letter and your manuscript will be scheduled for publication.

An invoice will be generated when your article is formally accepted. Please note, if your institution has a publishing partnership with PLOS and your article meets the relevant criteria, all or part of your publication costs will be covered. Please make sure your user information is up-to-date by logging into Editorial Manager at Editorial Manager® and clicking the ‘Update My Information' link at the top of the page. For questions related to billing, please contact billing support.

If your institution or institutions have a press office, please notify them about your upcoming paper to help maximize its impact. If they’ll be preparing press materials, please inform our press team as soon as possible -- no later than 48 hours after receiving the formal acceptance. Your manuscript will remain under strict press embargo until 2 pm Eastern Time on the date of publication. For more information, please contact onepress@plos.org.

Kind regards,

Esedullah Akaras

Academic Editor

PLOS One

Additional Editor Comments (optional):

Reviewers' comments:

Reviewer's Responses to Questions

**Comments to the Author**

1. If the authors have adequately addressed your comments raised in a previous round of review and you feel that this manuscript is now acceptable for publication, you may indicate that here to bypass the “Comments to the Author” section, enter your conflict of interest statement in the “Confidential to Editor” section, and submit your "Accept" recommendation.

Reviewer #1: All comments have been addressed

Reviewer #2: All comments have been addressed

**********

2. Is the manuscript technically sound, and do the data support the conclusions?

The manuscript must describe a technically sound piece of scientific research with data that supports the conclusions. Experiments must have been conducted rigorously, with appropriate controls, replication, and sample sizes. The conclusions must be drawn appropriately based on the data presented.

Reviewer #1: Yes

Reviewer #2: Yes

**********

3. Has the statistical analysis been performed appropriately and rigorously?

Reviewer #1: Yes

Reviewer #2: Yes

**********

4. Have the authors made all data underlying the findings in their manuscript fully available?

The PLOS Data policy requires authors to make all data underlying the findings described in their manuscript fully available without restriction, with rare exception (please refer to the Data Availability Statement in the manuscript PDF file). The data should be provided as part of the manuscript or its supporting information, or deposited to a public repository. For example, in addition to summary statistics, the data points behind means, medians and variance measures should be available. If there are restrictions on publicly sharing data—e.g. participant privacy or use of data from a third party—those must be specified.

Reviewer #1: Yes

Reviewer #2: Yes

**********

5. Is the manuscript presented in an intelligible fashion and written in standard English?

PLOS ONE does not copyedit accepted manuscripts, so the language in submitted articles must be clear, correct, and unambiguous. Any typographical or grammatical errors should be corrected at revision, so please note any specific errors here.

Reviewer #1: Yes

Reviewer #2: Yes

**********

6. Review Comments to the Author

Please use the space provided to explain your answers to the questions above. You may also include additional comments for the author, including concerns about dual publication, research ethics, or publication ethics. (Please upload your review as an attachment if it exceeds 20,000 characters)

Reviewer #1: Dear Authors,

All of my comments are addressed accordingly,

The manuscript has been improved.

Congratulations to your study

Reviewer #2: The authors have addressed all comments well. In my opinion, the article is suitable for publication in this journal.

**********

7. PLOS authors have the option to publish the peer review history of their article (what does this mean?). If published, this will include your full peer review and any attached files.

If you choose “no”, your identity will remain anonymous but your review may still be made public.

**Do you want your identity to be public for this peer review?**  For information about this choice, including consent withdrawal, please see our Privacy Policy.

Reviewer #1: **Yes:**  Hamed Esmaeili

Reviewer #2: **Yes:**  Mohamadreza Hatefi

**********

# Editor report: Acceptance letter

**DOI:** 10.1371/journal.pone.0342975.r004

**Authors:** Esedullah Akaras

PONE-D-25-60422R1

PLOS One

Dear Dr. Kurtoğlu,

I'm pleased to inform you that your manuscript has been deemed suitable for publication in PLOS One. Congratulations! Your manuscript is now being handed over to our production team.

At this stage, our production department will prepare your paper for publication. This includes ensuring the following:

* All references, tables, and figures are properly cited

* All relevant supporting information is included in the manuscript submission,

* There are no issues that prevent the paper from being properly typeset

You will receive further instructions from the production team, including instructions on how to review your proof when it is ready. Please keep in mind that we are working through a large volume of accepted articles, so please give us a few days to review your paper and let you know the next and final steps.

Lastly, if your institution or institutions have a press office, please let them know about your upcoming paper now to help maximize its impact. If they'll be preparing press materials, please inform our press team within the next 48 hours. Your manuscript will remain under strict press embargo until 2 pm Eastern Time on the date of publication. For more information, please contact onepress@plos.org.

You will receive an invoice from PLOS for your publication fee after your manuscript has reached the completed accept phase. If you receive an email requesting payment before acceptance or for any other service, this may be a phishing scheme. Learn how to identify phishing emails and protect your accounts at https://explore.plos.org/phishing.

If we can help with anything else, please email us at customercare@plos.org.

Thank you for submitting your work to PLOS ONE and supporting open access.

Kind regards,

PLOS ONE Editorial Office Staff

on behalf of

Dr. Esedullah Akaras

Academic Editor

PLOS One
